# FGF7/FGFR2–JunB signalling counteracts the effect of progesterone in luminal breast cancer

**DOI:** 10.1002/1878-0261.13274

**Published:** 2022-07-04

**Authors:** Kamil Mieczkowski, Kamila Kitowska, Marcin Braun, Barbara Galikowska‐Bogut, Monika Gorska‐Arcisz, Dominika Piasecka, Konrad Stawiski, Anna J. Zaczek, Dariusz Nejc, Radzisław Kordek, Hanna M. Romanska, Rafal Sadej

**Affiliations:** ^1^ Department of Molecular Enzymology and Oncology, Intercollegiate Faculty of Biotechnology University of Gdansk and Medical University of Gdansk Poland; ^2^ Department of Pathology, Chair of Oncology Medical University of Lodz Poland; ^3^ Department of Biostatistics and Translational Medicine Medical University of Lodz Poland; ^4^ Laboratory of Translational Oncology, Intercollegiate Faculty of Biotechnology Medical University of Gdansk Poland; ^5^ Department of Surgical Oncology Medical University of Lodz Poland

**Keywords:** ER, FGFR2, JunB, luminal breast cancer, PR, steroid hormones

## Abstract

We have recently demonstrated that fibroblast growth factor receptor 2 (FGFR2)‐mediated signalling alters progesterone receptor (PR) activity and response of oestrogen receptor α (ER)‐positive (ER+) breast cancer (BCa) cell lines to anti‐ER agents. Little is known about whether the crosstalk between ER and PR, shown to be modulated by the hormonal background, might also be affected by FGFR2. Here, PR‐dependent behaviour of ER+ BCa cells was studied in the presence of oestrogen (E2) and progesterone (P4) and/or FGF7. *In vitro* analyses showed that FGF7/FGFR2 signalling: (a) abolished the effect of P4 on E2‐promoted 3D cell growth and response to tamoxifen; (b) regulated ER and PR expression and activity; (c) increased formation of ER–PR complexes; and (d) reversed P4‐triggered deregulation of ER‐dependent genes. Analysis of clinical data demonstrated that the prognostic value of FGFR2 varied between patients with different menopausal status; that is, high expression of *FGFR2* was significantly associated with longer progression‐free survival (PFS) in postmenopausal patients, whereas there was no significant association in premenopausal patients. FGFR2 was found to positively correlate with the expression of JunB proto‐oncogene, AP‐1 transcription factor subunit (*JUNB*), an ER‐dependent gene, only in premenopausal patients. Molecular analyses revealed that the presence of JunB was a prerequisite for FGFR2‐mediated abrogation of P4‐induced inhibition of cell growth. Our results demonstrate for the first time that the FGF7/FGFR2–JunB axis abolishes the modulatory effects of PR on ER‐associated biological functions in premenopausal ER+ BCa. This may provide foundations for a better selection of patients for FGFR‐targeting therapeutic strategies.

## Graphical abstract

Oestrogen receptor α (ER) and progesterone receptor (PR), in the presence of steroid hormones, form a direct complex which regulates expression of a gene set associated with a good prognosis in luminal breast cancer (BCa). Here, we suggest that FGF7/FGFR2‐triggered signalling, in the premenopausal patients, engages JunB to the transcriptionally active complex resulting in a poor BCa patient outcome.

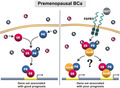

**Abbreviations**
BCa: breast cancer
DFS: disease‐free survival
E2: oestrogen
ER: oestrogen receptor α
FFPE: formalin‐fixed paraffin‐embedded
FGF7: fibroblast growth factor 7
FGFR2: fibroblast growth factor receptor 2
OHT: 4‐hydroxytamoxifen
OS: overall survival
P4: progesterone
PFS: progression‐free survival
PLA: proximity ligation assay
PR: progesterone receptor

## 1 Introduction

Oestrogen receptor α (ER), a key driver of development and growth of luminal breast cancer (BCa), is highly expressed in approximately 70% of all diagnosed BCa cases. The standard of care for ER‐positive (ER+) BCa patients involves drugs inhibiting ER activity (e.g. tamoxifen, fulvestrant), that significantly improves the outcome of the disease. However, *de novo* or acquired resistance to endocrine therapy still remains a major clinical problem [1, 2].

Around 50–60% of luminal BCa express progesterone receptor (PR). Since *PGR* encoding PR is an ER‐regulated gene [3], PR has been considered first and foremost as an indicator of ER transcriptional activity. However, recent evidence provides new insights into the biological role of PR in BCa, which alters the customary perception of its prognostic and predictive value [4]. Importantly, response to endocrine therapy and clinical outcome are more favourable in BCa patients who are ER+PR+ than those who are ER+PR− [5, 6]. In luminal BCa, the loss of PR is associated with disease progression towards a more aggressive, oestrogen‐independent and less responsive to anti‐ER treatment phenotype, which is often concurrent with an enhancement of receptor tyrosine kinases signalling [7, 8, 9]. Furthermore, functional crosstalk between ER and PR has been recently reported and shown that in the presence of both ligands, oestrogen and progesterone, PR associates with ER to redirect ER chromatin binding, which results in a unique, clinically favourable ER‐dependent genes expression profile [10, 11]. On the other hand, interaction between the two receptors and binding of the ER–PR complex to *CCND1* and *MYC* promoters are the prerequisites for progestin‐induced BCa cells proliferation [12]. This implies that the action of PR and its crosstalk with ER in luminal BCa is dependent on the ‘hormonal context’.

Activities of both receptors are known to be modulated also by paracrine stimuli derived from tumour microenvironment (TME). In particular, a functional association between FGF/FGFR2 signalling and regulation of steroid hormone receptors affecting responsiveness to endocrine therapy have been demonstrated in several studies [13, 14, 15]. For example, Giulianelli et al. identified FGFR2 as a mediator of hormone‐independent PR activation, which was induced by FGF2 secreted by cancer‐associated fibroblasts [16, 17]. We have recently shown that activation of FGF7/FGFR2 axis independently regulated phosphorylation and turnover of both ER and PR, leading to luminal BCa cells proliferation, anchorage‐independent growth and tamoxifen resistance *in vitro* [18, 19]. However, our subsequent analyses were not able to verify this mechanism in clinical material. Moreover, the data showed, in patients with ER+PR+ but not ER+PR− tumours, a positive association between FGFR2 and good prognosis [20]. This unexpected finding of a link between prognostic value of FGFR2 and PR status in luminal BCa may signify an involvement of FGFR2 signalling in the regulation of PR modulatory effects on ER‐dependent BCa. To test this hypothesis, we combined here cellular and molecular analyses with clinical studies to investigate a possible mechanism and biological consequence of the influence of FGFR2 on ER/PR activity in luminal BCa, in relation to the hormonal background.

## 2 Materials and methods

### 2.1 Cell culture and treatments

T47D and CAMA‐1 cell lines were purchased from DSMZ (Brunswick, Germany) and ATCC (Manassas, VA, USA), respectively. After reconstitution, all cells were passaged for a maximum of 2–3 months and regularly tested for mycoplasma contamination. T47D cells were grown in DMEM (Corning, NY, USA) and CAMA‐1 cells in MEM (Corning), both supplemented with 10% FBS (Biowest, Nuaillé, France), 100 U·mL^−1^ penicillin and 100 μg·mL^−1^ streptomycin (HyClone, Logan, UT, USA). For all treatments, the standard media were replaced with phenol red‐free DMEM (HyClone), serum‐free or, when appropriate, supplemented with 10% charcoal‐stripped FBS (Biowest). FGF7 (50 ng·mL^−1^, PeproTech, Rocky Hill, NJ, USA) was always applied with heparin sodium salt (50 ng·mL^−1^; Sigma‐Aldrich, St. Louis, MO, USA). 17β‐oestradiol (E2, oestrogen, 10 nm), progesterone (P4, 100 nm), 4‐hydroxytamoxifen (OHT, 1 μm), LY294002 (2 μm), SP600125 (10 μm), SU6656 (10 μm), UO126 (10 μm), and LiCl (20 mm) were purchased from Sigma‐Aldrich, AZD4547 (0.5 μm), SB216763 (10 μm), ABT‐199 (5 μm), SB202190 (10 μm) and Magnolol (10 μm) from Selleckchem (Houston, TX, USA) and BI‐D1870 (1 μm) from Axon Medchem (Groningen, the Netherlands).

### 2.2 Knock‐down of FGFR2 and JunB

T47D cells with knockdown of FGFR2 were derived with two different shRNA constructs. T47D FGFR2(−)^1^ cells were used in our previous studies [19] whereas T47D FGFR2(−)^2^ cells were established by lentiviral transfer of shRNA from Horizon discovery (RHS3979‐201732642; Dharmacon, Lafayette, CO, USA). T47D shJunB cells were generated with SMARTvector™ Human Lentiviral shRNA plasmids from Horizon discovery (V3SH11240; Dharmacon). The most potent and specific construct was chosen for the conducted experiments (clone ID: V3SVHS02_6201078). Stable knockdown clones were maintained in a medium supplemented with 0.2 μg·mL^−1^ puromycin (Sigma‐Aldrich). Stable silencing of FGFR2 and JunB was verified by immunoblotting before each set of experiments. In all experiments involving knockdown of FGFR2 or JunB, cells transfected with respective empty vectors were used as controls.

### 2.3 Three‐dimensional matrigel cultures

Cells were cultured in 3D matrigel as previously described [19]. Briefly, 1.5 × 10^3^ T47D or 2 × 10^3^ CAMA‐1 cells were resuspended in 40 μL (1 : 1 ratio) of growth factor reduced phenol red‐free Matrigel^®^ Basement Membrane Matrix (Corning) and cultured for 14 days. Media were replaced every 3 days. To evaluate cell growth, at least 70–100 colonies for each condition were measured using imagej software (National Institute of Health, Bethesda, MD, USA). Representative images were taken using ZEISS PrimoVert microscope (Oberkochen, Germany).

### 2.4 MTT proliferation assay

Cells were seeded into a 96‐well plate in triplicates and on the following day treatments were started. After 96 h, the 3‐(4,5‐dimethylthiazol‐2‐yl)‐2,5‐diphenyltetrazolium bromide (MTT, Sigma‐Aldrich) was added into each well (0.5 mg·mL^−1^) and incubated for 2 h at 37 °C. Then the medium was discarded and formazan crystals were dissolved in DMSO. The absorbance was measured at 590 nm.

### 2.5 Colony formation assay

1 × 10^3^ cells were seeded into a 12‐well plate. On the following day, the media were replaced with the media containing indicated hormones and/or FGF7, and/or specific inhibitors. Media were replaced every 3 days. After 10 days of culture, cells were washed with PBS, fixed with 4% paraformaldehyde, and stained with 0.4% crystal violet (Sigma‐Aldrich). Representative pictures were taken from three independent experiments.

### 2.6 Cell lysates and western blotting

Cells were grown to 70–80% confluence, scraped in ice‐cold PBS and lysed with Laemmli buffer (2× concentrated) supplemented with 2 mm PMSF, 10 μg·mL^−1^ aprotinin, 10 μg·mL^−1^ leupeptin, 5 mm EGTA, 1 mm EDTA, 2 mm Na_4_P_2_O_7_, 5 mm NaF, and 5 mm Na_3_VO_4_. An equal amount of protein (~ 20 μg) per lane was loaded, resolved in SDS/PAGE and transferred onto a nitrocellulose membrane. The membranes were blocked in 5% skimmed milk in TBS‐T and immunoblotted overnight with specific primary antibodies (described in Table S1) at 4 °C. Appropriate secondary antibodies conjugated with AlexaFluor^®^ 790 or AlexaFluor^®^ 680 (Jackson ImmunoResearch, West Grove, PA, USA) and Odyssey system (LI‐Cor, Lincoln, NE, USA) were used for the visualisation of detected proteins. Densitometry of bands representing detected proteins was done with image studio™ Software Ver 5.2 (Odyssey CLx, LI‐Cor).

### 2.7 Co‐immunoprecipitation

Fractionated cell nuclei from T47D cells treated for 1 h with E2 (CTR), E2 and P4 ± 30 min of FGF7 treatment were prepared using REAP method [21] from ~ 20 million cells. The nuclear fraction was lysed in 1% Triton X‐100 in PBS overnight at 4 °C. Then, the extracts were incubated with anti‐ERα antibodies (clone 1D5) coupled with protein A‐agarose beads. After 3–5 washings with 1% Triton X‐100 in PBS, co‐immunoprecipitated proteins were resuspended in 2× Laemmli buffer and analysed by western blotting.

### 2.8 Proximity ligation assay (PLA)

The Duolink™ *In Situ* PLA^®^ Technology (Sigma‐Aldrich) was used to detect an effect of the applied treatment on the ER–PR complex formation. Briefly, T47D and CAMA‐1 cells were starved for 24 h with serum‐free phenol red‐free DMEM, followed by 1 h E2 (10 nm, CTR) or E2 and P4 (100 nm) ± 30 min of FGF7 (50 ng·mL^−1^) treatment. Next, cells were fixed in 4% paraformaldehyde at room temperature (RT), permeabilised with 0.1% Triton X‐100 at 4 °C, blocked for 1 h at RT and incubated overnight with primary antibodies at 4 °C. All subsequent steps, that is, washing, incubation with secondary antibodies and detection were carried out according to the manufacturer's protocol. Slides were mounted with Duolink^®^ *In Situ* Mounting Medium with DAPI (Sigma‐Aldrich). ER–PR complexes were quantified as the number of detected dots per cell using imagej software. Representative images were taken using ZEISS AxioVert fluorescent microscope.

### 2.9 *In vitro* gene expression analyses

To analyse changes in the expression of ER‐dependent genes, T47D cells were serum‐starved in phenol red‐free medium and then treated with E2 (10 nm), E2 and P4 (100 nm), ± FGF7 (50 ng·mL^−1^) for 12 h. Total RNA was purified using the PureLink™ RNA Mini Kit (Invitrogen, Carlsbad, CA, USA). cDNA was synthesised using the Transcriptor cDNA First Strand Synthesis Kit (Roche, Basel, Switzerland) followed by an analysis of ER‐dependent genes using the RT2 Profiler Estrogen Receptor Signaling PCR Array (Qiagen, Hilden, Germany), according to the manufacturer's protocol. To analyse *IRS1* and *BCL2L1* expression (here used as biomarkers of ER transcriptional activity), T47D cells were serum‐starved in phenol red‐free medium, then treated for 6 h with E2 (10 nm) ± P4 (100 nm) ± FGF7 (50 ng·mL^−1^) and the following panel of inhibitors: LY294002 (2 μm), UO126 (10 μm), SB202190 (10 μm), BI‐D1870 (1 μm), SP600125 (10 μm), SU6656 (10 μm), ABT‐199 (5 μm) or Magnolol (10 μm). RNA isolation and cDNA synthesis were carried out as described above. For analysis of gene expression, the following TaqMan probes were used: *IRS1* (Hs00178563_m1), *BCL2L1* (Hs00236329_m1), as well as *ACTB* (Hs99999903_m1) and *GAPDH* (Hs02786624_g1), as reference genes. For qPCR reaction, TaqMan Universal PCR Master Mix (Applied Biosystem, Foster City, CA, USA) was used. Reactions were prepared in duplicates. Each plate contained a set of non‐template controls and controls for gDNA contamination. Gene expression was calculated using a modified ΔΔC approach [22].

### 2.10 Patient selection

Postoperative specimens from 246 treatment‐naïve patients diagnosed between 2012 and 2018 at the Regional Oncologic Centre of Copernicus Memorial Hospital, Lodz, Poland and at the Holycross Cancer Centre, Kielce, Poland with immunohistochemically determined ER+PR+ invasive breast carcinoma of no special type (IBC NST), were included in this study [20]. Clinical and pathological characteristics (in accordance with the WHO 2012 and 2019 classification of BCa [23]) of the group are presented in Table S2. Menopausal status was obtained from clinical records (menopause diagnosed after 12 months of amenorrhoea) [24]. If the information on menopausal status was lacking, patients of age of (a) ≤ 45 years and (b) ≥ 55 years were assigned as pre‐ and postmenopausal, respectively, and those between 45 and 55 years of age were excluded from the study [25]. The study was conducted in accordance with the Declaration of Helsinki and approved by the Local Research Ethics Committee (No. RNN/34/16/KE with amendment no. KE/15/21). Tumoural samples from patients recruited to this study were collected retrospectively post diagnosis (years 2012–2018) without any additive invasive procedures and at that time, no additional patient consent was obligatory.

### 2.11 Immunohistochemistry for FGFR2 expression

FGFR2 protein level was examined using immunohistochemistry and quantified in digitalized slides as described previously [20]. Immunohistochemical staining (IHC) was conducted on 5‐μm paraffin tumour sections using a mouse monoclonal anti‐FGFR2 antibody (H00002263‐M01; clone 1G3, Abnova, Taipei City, Taiwan). All slides were digitalized (Pannoramic 1000 Scanner, 3DHistech, Sysmex, Kobe, Japan) for quantification in 0–300 H‐score scale. Cases from 1st tercile of H‐score were regarded as FGFR2low and cases from 2nd and 3rd terciles were classified as FGFR2high.

### 2.12 Gene expression analyses in tumoural samples

For RNA quantification in clinical material, representative areas without necrosis, fibrosis or calcification were identified and dissected from formalin‐fixed paraffin‐embedded (FFPE) tumour samples. RNA was isolated using RNeasy FFPE Kit (Qiagen), which was followed by quality control on Tapestation 2200 (Agilent, Santa Clara, CA, USA). Quantification of RNA was done by Nanostring^®^ using nCounter PlexSet Expression analysis (Seattle, WA, USA) [20]. RNA counts were normalised using nSolver^®^ Analysis package (Nanostring). Four negative controls (normal mammary gland) and 16 internal controls (two samples of the same tumour; *n* = 5 and RNA measurement in duplicates, *n* = 3) were applied.

### 2.13 *In silico* analyses

Two independent external and publicly available datasets [METABRIC cohort from cBioPortal™; and non‐METABRIC cohort from The Cancer Genome Atlas (here called METABRIC and TCGA/non‐METABRIC, respectively)] were accessed for data on mRNA levels of *FGFR2* and *JUNB*, as well as available clinicopathological characteristics [25, 26, 27, 28]. The inclusion criteria were as follows: treatment‐naïve ER+/PR+ invasive breast carcinomas of no special type, reported menopausal status and follow‐up data. The Illumina Human v3 microarray mRNA data from cBioPortal™ METABRIC cohort were presented as *z*‐score values. Raw counts, harvested from TCGA, were divided by sample‐specific size factors determined by the median ratio of gene counts relative to geometric mean per gene, as described in DESeq2's median of ratios method [29]. Differential expression analysis utilised negative binomial modelling and hypothesis testing using the Wald test, as applied in DESeq2 method [30].

### 2.14 Statistical analysis

For clinical analyses, continuous data were presented as medians with interquartile ranges (IQR), whereas nominal data as numbers, followed by percentages in brackets. In the case of non‐normal distribution according to the Shapiro–Wilk test, continuous variables were compared by the Mann–Whitney *U*‐test for two groups or the Kruskal–Wallis test (AKW; with Conover‐Inman *post‐hoc* test) for multiple groups. For normal distribution, Student's *t*‐test or one−/two‐way block ANOVA (with Tukey's *post‐hoc* test) were used. Differences between categorical variables were evaluated using Pearson's chi‐squared test. The Spearman's rank correlation coefficients were calculated for correlations. Benjamini–Hochberg (BH) correction in case of multiple comparisons was applied. Disease‐free survival (DFS, the time from surgery to relapse, progression or death with censoring of living patients) and overall survival (OS, the time from diagnosis to death with censoring of living patients) were presented using Kaplan–Meier curves and compared using the Mantel–Cox log‐rank test unless noted otherwise. A multivariate analysis of OS and DFS was performed using Cox proportional hazard regression models. All *in vitro* data were presented as mean ± standard deviation (for experiments repeated at least three times) and Student's *t*‐test was used to compare the differences between two groups (using graphpad prism 8.0.1, GraphPad Software Inc., San Diego, CA, USA). Otherwise, the statistica 13.1 package (Dell Inc., Round Rock, TX, USA) was used. *P*‐values < 0.05 were considered as statistically significant.

## 3 Results

### 3.1 FGF7/FGFR2 abolishes progesterone‐induced inhibition of BCa cell growth via regulation of ER/PR expression and activation

**Fig. 1:**
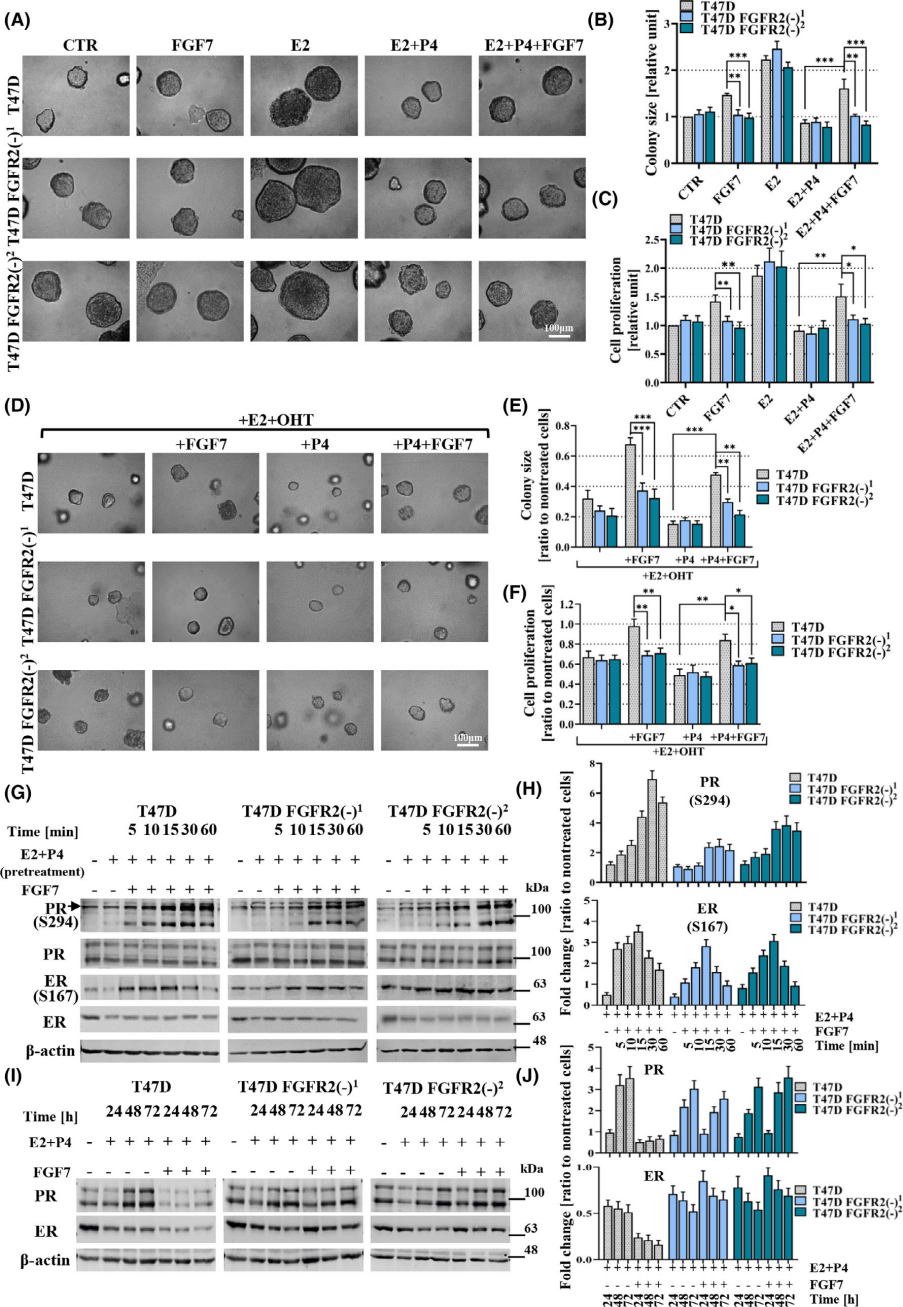
FGF7/FGFR2 abolishes progesterone‐induced inhibition of breast cancer (BCa) cell growth via regulation of ER/PR expression and activation. (A, B) T47D, T47D FGFR2(−)^1^ and T47D FGFR2(−)^2^ cells were cultured in 3D Matrigel for 14 days with E2 (oestrogen, 10 nm), and/or P4 (progesterone, 100 nm), and/or FGF7 (50 ng·mL^−1^). Representative images were taken (scale bar: 100 μm) (A) and colony size (B) was analysed using imagej software (relative to CTR/non‐treated wild‐type cells). (C) the effect of E2, and/or P4, and/or FGF7 treatment on proliferation of T47D, T47D FGFR2(−)^1^ and T47D FGFR2(−)^2^ cells was determined by MTT assay. Data for 3D Matrigel cultures and MTT assay are presented as means ± SD (*n* = 3), *P < 0.05, **P < 0.005, ***P < 0.001 by Student's *t‐*test. (D, E) T47D, T47D FGFR2(−)^1^ and T47D FGFR2(−)^2^ cells were cultured in 3D Matrigel for 14 days with E2, and/or P4, and/or FGF7 in the presence OHT (4‐hydroxytamoxifen, 1 μm). Representative images were taken (scale bar: 100 μm) (D) and colony size (relative to non‐treated wild‐type cells) (E) was analysed using imagej software. (F) Proliferation of T47D, T47D FGFR2(−)^1^ and T47D FGFR2(−)^2^ cells in the presence of E2 and OHT, and/or P4, and/or FGF7 was analysed by MTT assay. Data for 3D Matrigel cultures and MTT assay are presented as means ± SD (*n* = 3), *P < 0.05, **P < 0.005, ***P < 0.001 by Student's *t*‐test. (G, H) T47D, T47D FGFR2(−)^1^ and T47D FGFR2(−)^2^ cells were incubated for 24 h in the presence of E2 (10 nm) and P4 (100 nm) in serum‐free phenol red‐free medium and then treated with FGF7 (50 ng·mL^−1^) for 5, 10, 15, 30 and 60 min. PR and ER phosphorylation was analysed by western blotting and densitometry. (I, J) T47D and T47D FGFR2(−)^1^ and T47D FGFR2(−)^2^ cells were treated with E2 (10 nm) and P4 (100 nm) or with the combination of both steroid hormones with FGF7 (50 ng·mL^−1^) for 24, 48 and 72 h. PR and ER expression was evaluated by western blotting and densitometry. Densitometry data are presented as a ratio to control – Non‐treated cells, means ± SD (*n* = 3).

Following up on our recent study revealing an unexpected association between high expression of FGFR2 and good prognosis in ER+PR+ but not in ER+PR− BCa [20], here we employed an *in vitro* model (T47D and CAMA‐1 cell lines, both expressing ER, PR and FGFR2) to investigate the molecular mechanism underlying the functional link between FGFR2 and PR activity in ER‐dependent BCa. As previously demonstrated in both xenograft and primary ER+ BCa explants, progesterone (P4) induces inhibition of cell growth [10]. In order to assess the potential impact of FGFR2‐mediated signalling on the above effect, cells were treated with FGF7, an activating ligand for FGFR2, well‐documented for its role in physiology and pathophysiology of the mammary gland [31, 32, 33]. The specificity of FGF7 for FGFR2 was repeatedly demonstrated in our previous studies [18, 19]. As expected, P4 inhibited 3D growth in matrigel and cell proliferation of both T47D and CAMA‐1 cells. FGF7 was found to counteract P4‐induced cell growth inhibition (Fig. S1A–C,E–G). Involvement of FGFR2 in FGF7‐triggered effects was confirmed by knock‐down of FGFR2 in T47D cells. Stable silencing of FGFR2 by two specific shRNA constructs was confirmed (Fig. S1D) and proved not to affect the expression of other FGFRs. As expected, silencing of FGFR2 abolished the effect of FGF7 on T47D cells 3D growth and proliferation (Fig. S1A–C). Additionally, application of AZD4547 (a selective FGFR inhibitor) abrogated FGF7‐triggered effects on both T47D and CAMA‐1 cells 3D growth in matrigel (Fig. S1H,I,K,L). The effects of FGFR2 silencing and AZD4547 were further confirmed in T47D cells colony formation assay (Fig. S1J). To mimic the premenopausal hormonal environment of *in vivo* BCa, we applied oestrogen (E2) together with P4 and analysed the role of FGF7 in such a setup. The impact of FGFR2 activity on cell response to E2 (as a single treatment) has been already demonstrated [34, 35]. Confirming previous findings [10], P4 was found to inhibit E2‐promoted 3D growth of T47D and CAMA‐1 cells as well as proliferation of T47D cells, whereas FGF7 treatment strongly reduced this suppressive effect (Fig. 1A, upper panel, Fig. 1C and Fig. S2A, upper panel). FGF7‐induced promotion of growth, proliferation and colony formation were not observed in T47D FGFR2(−) cells (Fig. 1A–C and Fig. S3D, middle panel), which confirms that FGFR2 mediates FGF7‐regulated steroid hormone‐dependent BCa growth. Moreover, AZD4547 was shown to abrogate the effects of FGF7 in the presence of steroid hormones (Figs S2A–C and S3A–D). Moreover, AZD4547 also affected the cell response to E2 which might be associated with its effect on other FGFRs. For instance, FGFR1 was previously described as a modulator of ER activity [36, 37]. Taken together, these results indicate an involvement of FGF7/FGFR2 signalling in the regulation of cell response to both steroid hormones, characteristic for premenopausal patients. As demonstrated by Mohammed et al. [10], in xenograft models, progesterone enhanced the activity of tamoxifen in BCa. Following this observation, we next assessed a possible involvement of FGFR2 in cell response to 4‐hydroxytamoxifen (OHT, an active metabolite of tamoxifen) in the presence of both E2 and P4. FGF7 stimulation was found to antagonise the negative effect of tamoxifen on the growth and proliferation of T47D and CAMA‐1 cells in the medium supplemented with E2 ± P4 (Fig. 1D–F and Fig. S2D–F), and this was abolished by silencing of FGFR2 (Fig. 1D–F) or FGFRs inhibition (Figs S2D–F and S3E–H).

To further identify the molecular mechanism underlying FGF7/FGFR2‐mediated regulation of hormone‐dependent BCa cell growth, activation of ER and PR upon FGF7 treatment in the presence of E2 and P4 was studied. The results showed that in the presence of both steroid hormones, FGF7 induced phosphorylation of ER and PR in T47D (Fig. 1G, left panel) and CAMA‐1 (Fig. S4A,B) cells. The pattern of ER S167 phosphorylation (required for activation of ER; the highest peak at 10–15 min) was similar in both cell lines, whereas the kinetics of PR S294 phosphorylation (required for hyperactivation followed by proteasomal degradation of PR) varied, with the highest peak at 30 and 15 min, in T47D and CAMA‐1 cells, respectively (Fig. 1G, left panel, Fig. 1H and Fig. S4A,B). FGFR2 silencing in T47D cells significantly modified FGF7‐triggered PR and ER phosphorylation (Fig. 1G,H).

To investigate whether FGF7/FGFR2‐triggered signalling affects the stability of ER and PR, T47D, their two variants with FGFR2 knockdown and CAMA‐1 cells were incubated with E2 and P4 ± FGF7 for 24, 48 and 72 h. It was found that E2 + P4 treatment induced a drop in PR expression after 24 h, which was followed by an increase in PR level after 48 and 72 h (Fig. 1I, left panel and Fig. S4C,D). This might reflect initially the P4‐triggered turnover of PR, followed by a rise of its expression in response to E2. In both tested cell lines, ER levels decreased and remained unchanged during hormonal treatment (Fig. 1I, left panel and Fig. S4C,D). This may explain the decrease of ER phosphorylation observed after 24 h of pretreatment with E2 (Fig. 1G,H and Fig. S4A,B). Importantly, FGF7/FGFR2 activity in the presence of E2 and P4 led to a greater and stable decrease of both ER and PR in T47D and CAMA‐1 cells (Fig. 1I, left panel and Fig. S4C,D), which was abolished by knock‐down of FGFR2 in T47D cells (Fig. 1I,J).

### 3.2 FGF7/FGFR2 alters an effect of progesterone on formation of the ER‐PR complex and transcriptional activity of ER

**Fig. 2:**
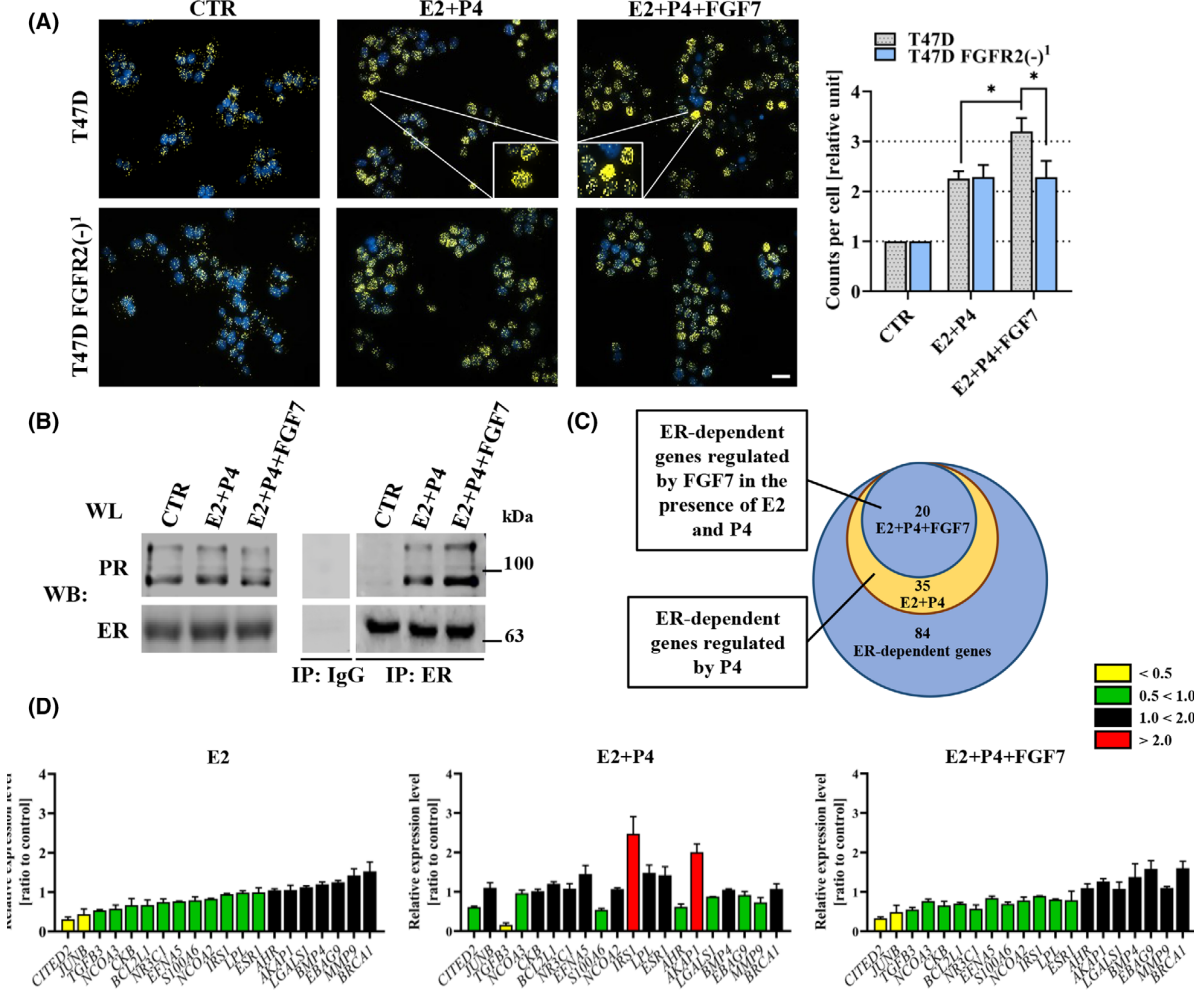
FGF7/FGFR2 alters the effect of progesterone on formation of the ER–PR complex and transcriptional activity of ER. (A) the number of ER‐PR complexes in T47D and T47D FGFR2(−)^1^ cells upon E2 (CTR; oestrogen, 10 nm), E2 with P4 (progesterone, 100 nm) ± FGF7 (50 ng·mL^−1^) treatment was analysed using proximity ligation assay. Representative fluorescent microscopy images were taken (scale bar: 20 μm). Protein–protein interactions were quantified using imagej software and presented as a mean number of immunofluorescent dots per cell (±SD; *n* = 3), *P < 0.05 by Student's *t*‐test. (B) Nuclear extracts from T47D cells treated for 1 h with E2 (CTR), E2 and P4 ± 30 min with FGF7 were incubated with anti‐ERα antibodies (clone 1D5) coupled with protein A‐agarose beads. IgG was used as an immunoprecipitation negative control. Co‐immunoprecipitated proteins were analysed by western blotting, representative immunoblot is shown (*n* = 3). (C) Venn diagram showing the number of ER‐dependent genes the expression of which was altered by P4 and further restored by FGF7 treatment. (D) Analysis of mRNA expression changes of ER‐dependent genes in T47D cells upon E2 (10 nm), E2 with P4 (100 nm) ± FGF7 (50 ng·mL^−1^), using the RT2 profiler Oestrogen receptor Signalling PCR Array. Data are shown as a ratio to control, means ± SD (*n* = 3).

The physical and functional interdependence between ER and PR has been already well‐documented [10, 12, 38, 39]. Thus, FGF7/FGFR2 signalling was further investigated for possible involvement in the formation of the ER–PR complex. T47D and T47D FGFR2(−) cells were serum‐starved which was followed by treatment with E2 and P4 ± FGF7. Using the proximity ligation assay, a number of ER–PR complexes were analysed. As previously reported [10], the E2 and P4 co‐treatment induced formation of ER–PR complexes in T47D and CAMA‐1 cells. Importantly, this was further enhanced by FGF7 (Fig. 2A, upper panel and Fig. S5), which was abolished by knock‐down of FGFR2 (Fig. 2A, bottom panel). FGF7‐enhanced formation of ER–PR complexes in response to E2 + P4 was confirmed with co‐immunoprecipitation experiments in T47D cells (Fig. 2B).

To define molecular consequences of FGF7‐induced ER–PR interaction, we analysed a significance of FGF7 in the regulation of P4‐modulated ER transcriptional activity. RT^2^ Profiler PCR Array was used to detect the expression of 84 ER‐dependent genes. Expression of 35 of these genes was found to be altered by P4 (Fig. 2C). Moreover, in the presence of both steroid hormones, FGF7 reverted P4‐induced deregulation of 20 out of 35 of these genes back to the pattern characteristic for treatment only with E2 (Fig. 2C,D). This confirms the functional antagonism of FGF7 and P4 in the regulation of ER transcriptional activity.

### 3.3 Good prognostic effect of high expression of FGFR2 is lost in premenopausal BCa patients

The level of circulating steroid hormones, that is, oestrogens and progesterone that activate ER and PR signalling in BCa, depends on the hormonal status/age of the patients. While in premenopausal patients the levels of both are adequate, in postmenopausal women only a small amount of oestrogens are being produced by adipose tissue and there is a lack of progesterone [40]. To assess the postulated impact of hormonal background (hereafter called ‘the menopausal status’) on the previously shown good prognostic effect of FGFR2 in ER+PR+ BCa [20], only treatment‐naïve patients were included in the study and assessment of FGFR2 expression evaluated at both gene and protein level.

**Fig. 3:**
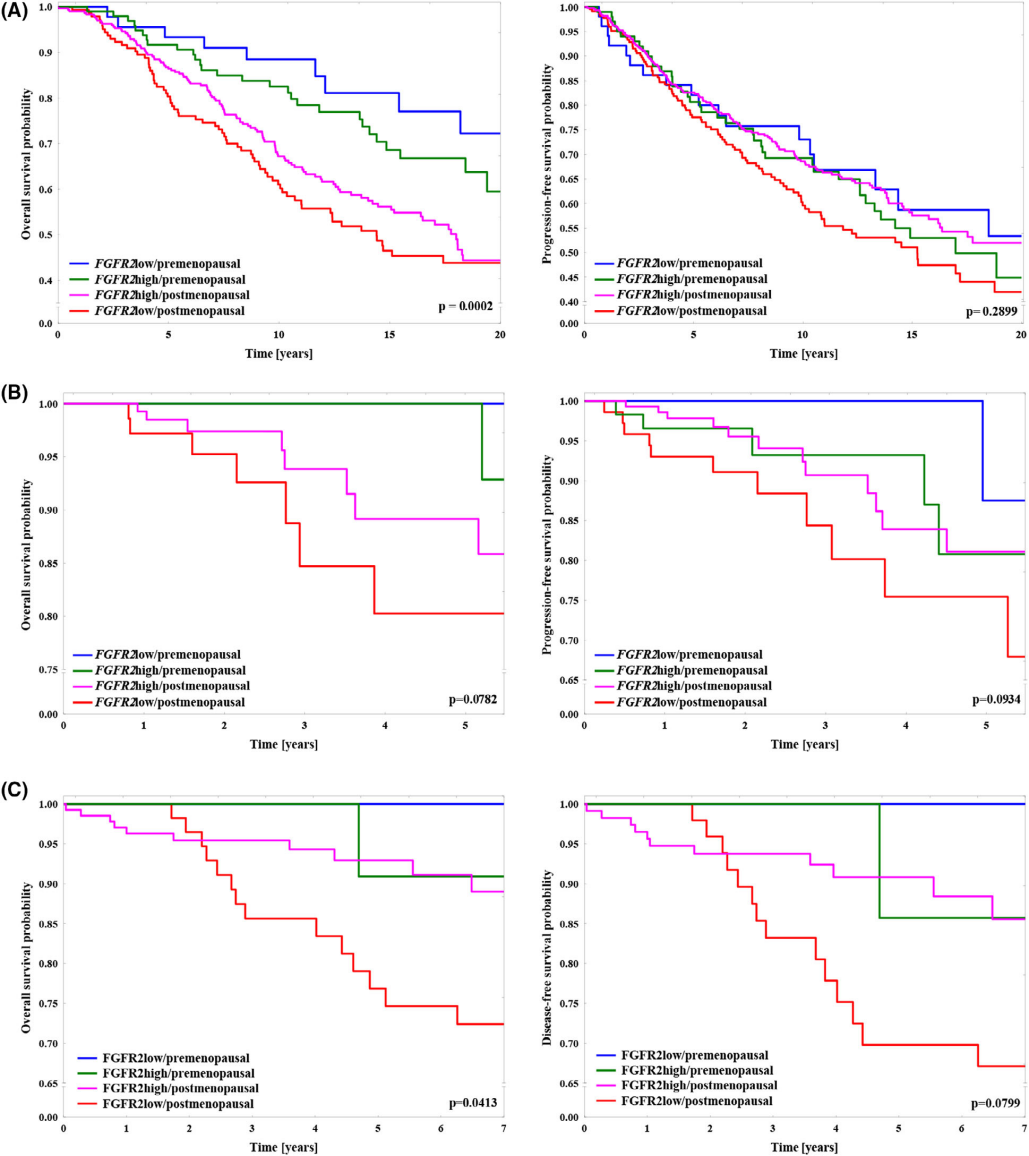
Prognostic effect of *FGFR2* mRNA level is opposite in premenopausal and postmenopausal breast cancer (BCa) patients. (A) Kaplan–Meier curve for overall survival (OS) and progression‐free survival (PFS) probability in data from the METABRIC cohort (*FGFR2* status determined by 1st tercile of *FGFR2* mRNA), *P*‐values were calculated using the mantel‐cox log‐rank test: For OS *P* = 0.0002 for the entire OS model, *P* = 0.0989 (HR: 0.83 (95%CI: 0.62–1.11)) for the difference between *FGFR2*high (182 censored and 120 uncensored cases) vs. *FGFR2*low (73 censored and 70 uncensored cases) in postmenopausal patients and *P* = 0.2746 (HR: 1.56 (95%CI: 0.74–3.33)) for the difference between *FGFR2*high (72 censored and 28 uncensored cases) vs. *FGFR2*low (37 censored and nine uncensored cases) in premenopausal; for PFS *P* = 0.2899 for the entire PFS model, *P* = 0.0386 (HR: 0.76 (95%CI: 0.59–0.99)) for the difference between *FGFR2*high (293 censored and 153 uncensored cases) vs. *FGFR2*low (126 censored and 98 uncensored cases) in postmenopausal patients and *P* = 0.5984 (HR: 1.16 (0.67–2.02)) for the difference between *FGFR2*high (61 censored and 41 uncensored cases) vs. *FGFR2*low (33 censored and 18 uncensored cases) in premenopausal patients. (B) Kaplan–Meier curve for OS and PFS probability in data from the TCGA/non‐METABRIC cohort (*FGFR2* status determined by 1st tercile of *FGFR2* mRNA), *P*‐values were calculated using the mantel‐cox log‐rank test: For OS *P* = 0.0782 for the entire OS model, *P* = 0.2413 (HR: 0.57 (95%CI: 0.21–1.58)) for the difference between *FGFR2*high (144 censored and eight uncensored cases) vs. *FGFR2*low (66 censored and seven uncensored cases) in postmenopausal patients, HR for the difference between *FGFR2*high (58 censored and one uncensored cases) vs. *FGFR2*low (28 censored and 0 uncensored cases) in premenopausal patients was not calculable (not enough cases to perform analyses); for PFS *P* = 0.0934 for the entire PFS model, *P* = 0.0551 (HR: 0.51 (95%CI: 0.22–1.15)) for the difference between *FGFR2*high (144 censored and 12 uncensored cases) vs. *FGFR2*low (62 censored and 11 uncensored cases) in postmenopausal patients and *P* = 0.2373 (HR: 2.71 (0.32–23.32)) for the difference between *FGFR2*high (55 censored and five uncensored cases) vs. *FGFR2*low (27 censored and one uncensored cases) in premenopausal patients. (C) Kaplan–Meier curves for OS and disease‐free survival (DFS) probability of patients stratified according to FGFR2 (protein) and menopausal status (FGFR2 expression determined by 1st tercile of FGFR2 protein H‐score). The good prognosis, in terms of both OS and DFS, was observed in the FGFR2high/postmenopausal (128 censored and 10 uncensored cases for OS; 104 censored and 11 uncensored cases for DFS) patients, which differed significantly when compared with the FGFR2low/postmenopausal (47 censored and 14 uncensored cases for OS; 39 censored and 14 uncensored cases for DFS) group; HR (95%CI) for OS = 0.37 (0.16–0.83), *P* = 0.0097 and DFS = 0.40 (0.18–0.88), *P* = 0.0157. In the premenopausal subgroups, the FGFR2 prognostic value was not calculable (not enough cases to perform analyses; 25 censored and one uncensored cases for OS, and 20 censored and one uncensored cases for DFS in FGFR2high subgroup; six censored and 0 uncensored cases for OS; four censored and 0 uncensored cases for DFS in FGFR2low subgroup), *P*‐values were calculated using the mantel‐cox log‐rank test.

Evaluation of a prognostic value of *FGFR2* mRNA was based on an analysis of clinical data of *n* = 825 patients included from the METABRIC dataset, and *n* = 338 from the TCGA/non‐METABRIC cohort (see inclusion criteria in Methods). The results from the METABRIC dataset showed that the prognostic value of *FGFR2* mRNA was dependent on the menopausal status of the patients. In premenopausal women, *FGFR2*high mRNA was not significantly associated with shorter both overall and progression‐free survival (HR for OS: 1.56 (95%CI: 0.74–3.33), *P* = 0.2746; HR for PFS: 1.16 (95%CI: 0.67–2.02), *P* = 0.5984), whereas the correlation of *FGFR2*high mRNA with good prognosis was maintained in postmenopausal group (HR for OS with *FGFR2*high mRNA as reference = 0.83 (95%CI: 0.62–1.11), *P* = 0.0989; HR for PFS: 0.76 (95%CI: 0.59–0.99), *P* = 0.0386; *P* = 0.0002 for the entire model for OS and *P* = 0.2899 for PFS; Fig. 3A). These results were additionally validated in the TCGA/non‐METABRIC dataset. Herein, high transcript level of *FGFR2* tended to associate with good prognosis in postmenopausal group of ER+PR+ BCa patients (HR for PFS: 0.51 (95%CI: 0.22–1.15), *P* = 0.0551; HR for OS: 0.57 (95%CI: 0.21–1.58), *P* = 0.2413), whereas these effects were abolished in premenopausal women (HR for PFS: 2.71 (95%CI: 0.32–23.32), *P* = 0.2373; HR for OS was not calculable; Fig. 3B).

In a next step, an assessment of FGFR2 protein level in relation to the clinicopathological characteristics, including the menopausal status, was carried out in tissue samples from 246 treatment‐naïve patients with ER+PR+ BCa. According to the FGFR2 expression and the menopausal status (available for 231 (93.9%) patients) the analysed cohort was divided into four subgroups: (a) FGFR2low/premenopausal (*n* = 6/231, 2.6%); (b) FGFR2low/postmenopausal (*n* = 61/231, 26.4%); (c) FGFR2high/premenopausal (*n* = 26/231, 11.3%); and (d) FGFR2high/postmenopausal (138/231, 59.7%; Table S2). Expression of FGFR2 did not significantly differ between pre‐ and postmenopausal patients (H‐scores of 120 (42–217) vs. 95 (21–195), *P* = 0.2626, Fig. S6). Regardless of the FGFR2 status, premenopausal vs. postmenopausal patients were more frequently linked with lymph node metastases (*P* = 0.023), presence of DCIS component (*P* = 0.001) and chemotherapy in the adjuvant setting (*P* = 0.006). No significant differences between the subgroups were observed for Ki67 index, *HER2* amplification, tumour size or staging (Table S2). Although the numerical imbalance (pre‐ vs. postmenopausal cases) between the subgroups did not allow for the generation of statistically significant data, the results clearly indicate an association of the prognostic values of FGFR2 with the menopausal status of the patients, thus confirming the findings of *in silico* analyses (Fig. 3C). Taken together, analyses of clinical data support our initial hypothesis and *in silico* findings that FGFR2 may regulate BCa cell response to steroid hormones.

### 3.4 FGFR2 correlates with *JUNB* in premenopausal BCa

The results described above (Paragraph 3.2) have led to the identification of a panel of ER‐dependent genes modulated by FGF7 in the presence of both steroid hormones, that was further used in the Nanostring‐based RNA quantitative analysis of BCa samples to detect molecular candidate/s of biologically meaningful association with FGFR2. To extrapolate the *in vitro* results to clinical setting, analyses of correlations between the expression of FGFR2 (protein) and mRNA of individual genes have been carried out and revealed that the expression of nine of them significantly correlated with FGFR2 (protein) in the whole group of patients (Table S3). Stratification of patients according to their menopausal status showed that only median expression of *ESR1* was significantly distinguishing between pre‐ and postmenopausal groups (Table S4). Further analyses within the specific menopausal subgroups of expression of selected genes in relation to that of FGFR2 disclosed a distinctly high positive correlation between *JUNB* and FGFR2 in the premenopausal women (*R* = 0.5, *P* = 0.0034, BH‐corrected *P* = 0.0719; Table 1A) and modestly positive correlations between *BCL2L1*, *MMP9*, *BRCA1* and FGFR2 in the postmenopausal cohort of patients (Table 1B).

**Table 1:** Correlation between FGFR2 protein levels and mRNA levels of *in vitro* specified biomarkers of ER activity in clinical material. Analysis of correlation between FGFR2 protein levels and mRNA levels of *in vitro* specified biomarkers of ER activity (based on RT2 Oestrogen Receptor Signalling PCR Array) in clinical material. Spearman correlation coefficients with raw *P*‐values and Benjamini–Hochberg (BH) corrected *P*‐values are presented. Genes are arranged according to significance level of correlations. (A) Premenopausal tumours (*n* = 32), (B) Postmenopausal tumours (*n* = 199).

Correlation between FGFR2 protein [H‐score] levels with mRNA [log2] levels of:	Correlation coefficient (*R*)	Raw *P*‐value	BH‐corrected, *P*‐value
A			
*JUNB*	0.50	0.0034	0.0719
*TGFB3*	0.40	0.0235	0.2472
*S100A6*	0.37	0.0353	0.2472
*CITED2*	0.30	0.0944	0.4958
*NR3C1*	0.23	0.21	0.6563
*LPL*	0.21	0.2412	0.6563
*CCL2*	0.21	0.2421	0.6563
*BCL2L1*	0.21	0.2500	0.6563
*IRS1*	0.18	0.3321	0.7749
*MMP9*	−0.15	0.4126	0.8664
*EBAG9*	−0.11	0.5529	0.9191
*LGALS1*	0.10	0.5733	0.9191
*BMP4*	−0.09	0.6298	0.9191
*AHR*	−0.08	0.6491	0.9191
*BMP7*	−0.07	0.6936	0.9191
*BRCA1*	−0.07	0.7002	0.9191
*EFNA5*	0.05	0.7817	0.9634
*NCOA3*	0.02	0.8922	0.9634
*AKAP1*	0.02	0.9033	0.9634
*ESR1*	−0.02	0.9175	0.9634
*CKB*	0.00	0.9849	0.9849
B			
*BCL2L1*	0.24	0.0006	0.0124
*MMP9*	0.22	0.0012	0.0125
*BRCA1*	0.19	0.0051	0.0361
*IRS1*	0.18	0.0099	0.0520
*S100A6*	0.16	0.0191	0.0710
*AHR*	0.16	0.0203	0.0710
*CKB*	0.15	0.0288	0.0810
*EFNA5*	0.15	0.0308	0.0810
*ESR1*	0.14	0.04	0.0933
*NCOA3*	0.12	0.0769	0.1614
*JUNB*	0.11	0.1038	0.1982
*LGALS1*	0.11	0.1193	0.2088
*CCL2*	0.09	0.1917	0.3097
*LPL*	0.08	0.2128	0.3191
*CITED2*	0.07	0.2883	0.4036
*BMP4*	0.06	0.3372	0.4165
*NR3C1*	0.06	0.3372	0.4165
*EBAG9*	−0.06	0.3899	0.4549
*TGFB3*	0.04	0.503	0.5559
*AKAP1*	0.04	0.5446	0.5705
*BMP7*	−0.04	0.5705	0.5705

### 3.5 MAPK/JNK signalling pathway mediates FGF7‐regulated ER transcriptional activity and hormone‐dependent BCa cell growth

**Fig. 4:**
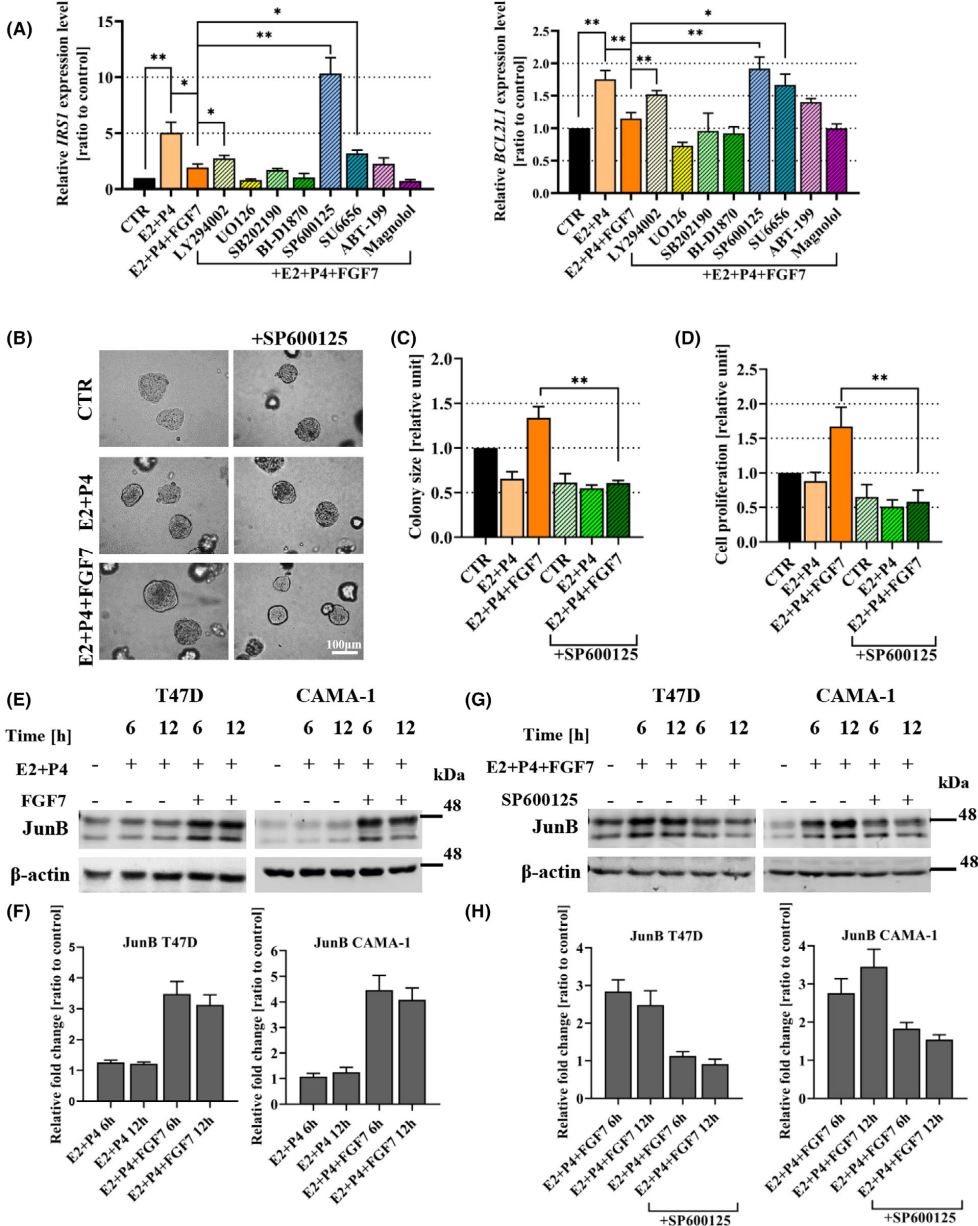
MAPK/JNK signalling pathway mediates FGF7‐regulated ER transcriptional activity and hormone‐dependent breast cancer (BCa) cell growth. (A) T47D cells were serum‐starved in phenol red‐free medium and treated with E2 (oestrogen,10 nm) and P4 (progesterone, 100 nm), or FGF7 (50 ng·mL^−1^) with both steroid hormones ± LY294002 (2 μm), or UO126 (10 μm), or SB202190 (10 μm), or BI‐D1870 (1 μm), or SP600125 (10 μm), or SU6656 (10 μm), or ABT‐199 (5 μm), or Magnolol (10 μm) for 6 h. relative expression of *IRS1* and *BCL2L1* (used as biomarkers of ER transcriptional activity) was analysed by RT‐qPCR. Each bar represents ratio to control, means ± SD (*n* = 3), *P < 0.05, **P < 0.005 by Student's *t*‐test. (B, C) T47D cells were cultured in 3D Matrigel for 14 days with E2 (10 nm) and P4 (100 nm), ± FGF7 (50 ng·mL^−1^), ± SP600125 (10 μm). Representative images were taken (scale bar: 100 μm) (B) and colony size (relative to CTR/non‐treated cells) (C) was analysed using imagej software. Data are presented as means ± SD (*n* = 3), **P < 0.005 by Student's *t*‐test. (D) the effect of E2 and P4, ± FGF7, ± SP600125 treatment on proliferation of T47D cells was analysed by MTT assay. Data are presented as means ± SD (*n* = 3), **P < 0.005 by Student's *t*‐test. (E, F) T47D and CAMA‐1 cells were treated with E2 (10 nm) and P4 (100 nm) ± FGF7 (50 ng·mL^−1^) for 6 and 12 h. JunB expression was analysed by western blotting and densitometry. Densitometry data are presented as a ratio to control – Non‐treated cells, means ± SD (*n* = 3). (G, H) T47D and CAMA‐1 cells were treated with E2 (10 nm), P4 (100 nm) and FGF7 (50 ng·mL^−1^) ± SP600125 (10 μm) for 6 and 12 h. JunB expression was analysed by western blotting and densitometry. Densitometry data are presented as a ratio to control – Non‐treated cells, means ± SD (*n* = 3).

The functional interdependence between FGFR2, ER and PR suggested by clinical analyses was further investigated at the molecular level in the *in vitro* model. First, to identify a signalling pathway involved in FGF7/FGFR2‐dependent regulation of ER transcriptional activity upon E2 + P4 treatment, RT‐qPCR was carried out for *IRS1* and *BCL2L1* (demonstrated in a pilot experiment to exhibit the most prominent differences in expression at 6 h of treatment with E2 + P4; Fig. S7A). The expression level of *IRS1* and *BCL2L1* was significantly increased in response to E2 and P4 treatment, which was strongly impaired by FGF7 (Fig. 4A). Specific inhibitors of signalling pathways activated by FGFR signalling were then applied, including LY294002 (PI3K/Akt), UO126 (ERK1/2), SB202190 (p38), BI‐D1870 (RSK1‐3), SP600125 (JNK), SU6656 (Src), ABT‐199 (Bcl‐2) and Magnolol (NFκB) [41, 42]. The results showed that PI3K/Akt, Src and JNK might be involved in FGF7‐regulated transcriptional activity of ER in the presence of E2 and P4, with the most significant effect exerted by JNK inhibitor (SP600125; Fig. 4A). To verify these results, FGF7/FGFR2‐driven hormone‐dependent cell growth was analysed. The data showed that SP600125 completely abrogated FGF7 stimulatory effects in the presence of E2 and P4 (Fig. 4B–D and Fig. S7B), whereas LY294002 and SU6656 effects were less conspicuous (Fig. S7C, D), confirming that JNK signalling pathway might be a major mediator of FGF7/FGFR2 signalling targeting ER activity in the hormonal environment specific for premenopausal BCa. This is in agreement with clinical analyses demonstrating in premenopausal patients a strong correlation of FGFR2 with *JUNB*, the member of JUN family transcription factors, a well‐known substrate of JNK kinases (Table 1A) [43]. To confirm mechanistically this link, expression of JunB (protein) was analysed in BCa cell lines upon E2 and P4 ± FGF7 treatment. The results showed that in conditions mimicking premenopausal status, FGF7 promoted JunB expression in T47D and CAMA‐1 cells (Fig. 4E,F). Importantly, inhibition of JNK activity (by SP600125) reduced the effect of FGF7‐inducing expression of JunB in both cell lines (Fig. 4G,H). These results support clinical observations of FGFR2/JunB relationship and indicate that in the presence of E2 and P4, JunB is a downstream effector of FGF7/FGFR2‐JNK signalling pathway.

### 3.6 JunB is required for FGF7‐triggered abrogation of progesterone‐induced inhibition of BCa cell growth

**Fig. 5:**
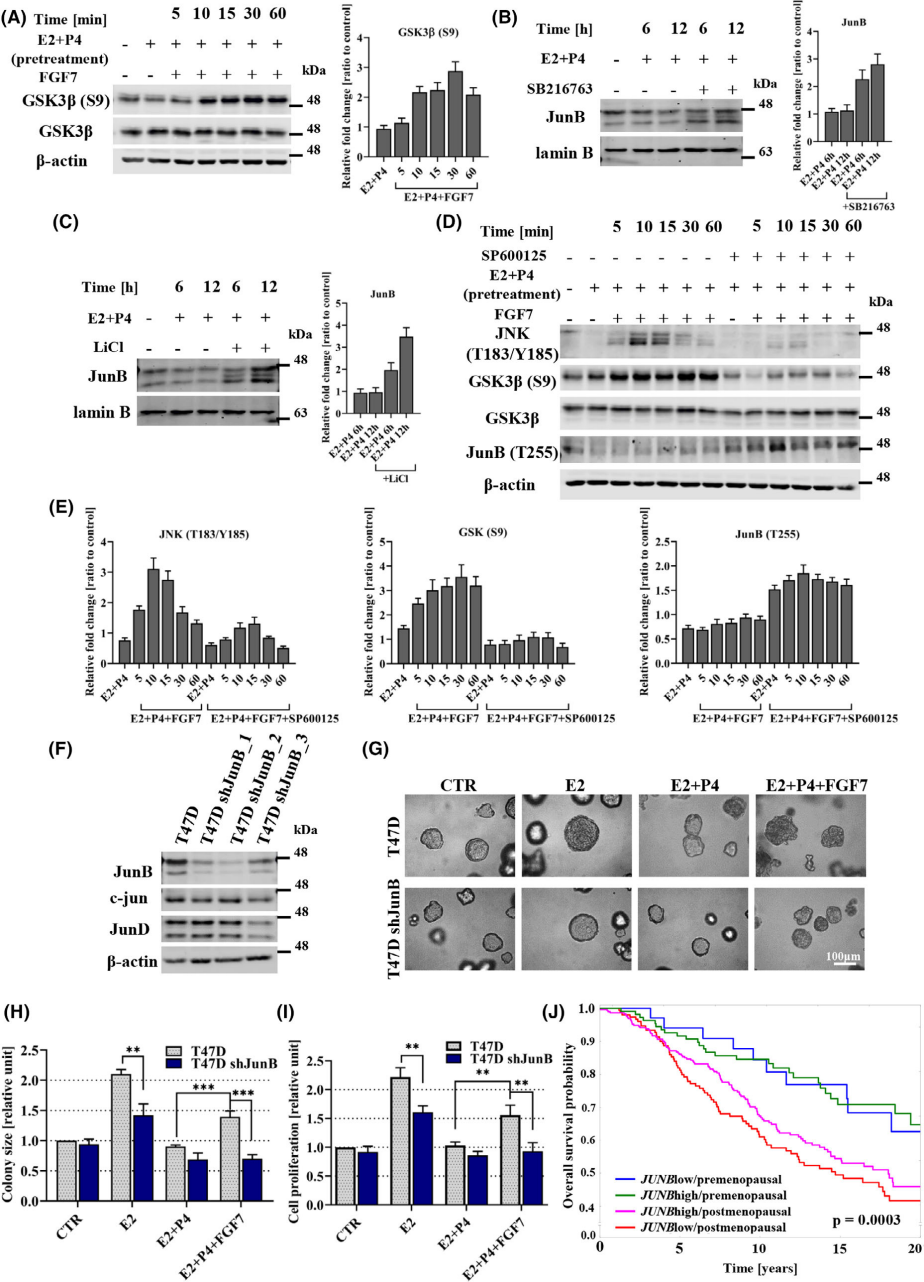
JunB is required for FGF7‐triggered abrogation of the negative effect of P4 on breast cancer (BCa) cell growth. (A) T47D cells were pre‐incubated for 24 h with E2 (oestrogen, 10 nm) and P4 (progesterone, 100 nm) in serum‐free phenol red‐free medium and then treated with FGF7 (50 ng·mL^−1^) for 0–60 min. GSK3β serine 9 phosphorylation (which inhibits its activity) was analysed by western blotting and densitometry. Densitometry data are presented as a ratio to control – Non‐treated cells, means ± SD (*n* = 3). (B, C) T47D cells were treated with E2 and P4 ± SB216763 (10 μm) (B) or LiCl (20 mm) (C) for 6 and 12 h. expression of JunB was analysed by western blotting and densitometry. Densitometry data are presented as a ratio to control – Non‐treated cells, means ± SD (*n* = 3). (D, E) T47D cells were incubated for 24 h with serum‐free phenol red‐free medium supplemented with E2 (10 nm) and P4 (100 nm) ± SP600125 (10 μm), followed by FGF7 (50 ng·mL^−1^) treatment for 0–60 min. JNK, GSK3β and JunB phosphorylation were analysed by western blotting and densitometry. Densitometry data are presented as a ratio to control – Non‐treated cells, means ± SD (*n* = 3). (F) Specificity and efficiency of JunB silencing with three shRNA constructs in T47D cells was evaluated by western blot analysis. (G, H) T47D and T47D shJunB (shJunB_2) cells were cultured in 3D Matrigel for 14 days with E2 (10 nm) ± P4 (100 nm), or with FGF7 (50 ng·mL^−1^) in the presence of both steroid hormones. Representative images were taken (scale bar 100 μm) (G) and colony size (relative to CTR/non‐treated wild‐type cells) (H) was analysed using imagej software. Data are presented as means ± SD (*n* = 3), **P < 0.005, ***P < 0.001 by Student's *t*‐test. (I) the effect of E2, E2 and P4 ± FGF7 on proliferation of T47D and T47D shJunB cells was analysed by MTT assay. Data are presented as means ± SD (*n* = 3), **P < 0.005 by Student's *t*‐test. (J) Kaplan–Meier curve for overall survival (OS) probability in data from the METABRIC cohort (*JUNB* status determined by 1st tercile of *JUNB* mRNA), *P*‐values were calculated using the mantel‐cox log‐rank test: *P* = 0.0003 for the entire model, *P* = 0.9393 (HR: 0.95 (95%CI: 0.46–1.96) for the difference between *JUNB*high (85 censored and 27 uncensored cases) vs. *JUNB*low (24 censored and 10 uncensored cases) in premenopausal and *P* = 0.1366 (HR: 0.83 (95%CI: 0.62–1.67) for the difference between *JUNB*high (177 censored and 115 uncensored cases) vs. *JUNB*low (78 censored and 75 uncensored cases) in postmenopausal patients.

The mechanism of regulation of JunB expression, identified so far, involves GSK3β‐mediated phosphorylation of JunB T255, which is followed by its ubiquitination driven by FBW7 E3 ubiquitin ligase and subsequent proteasomal degradation [44, 45]. Our data showed that in the presence of E2 and P4, FGF7 induced GSK3β‐S9 phosphorylation in T47D cells, known to be responsible for inhibition of GSK3β activity (Fig. 5A) [46]. Additionally, application of the inhibitors of GSK3β, that is, SB216763 and LiCl [47, 48], in the presence of E2 and P4, led to the increase of JunB expression in T47D and CAMA‐1 cells (Fig. 5B,C and Fig. S8A,B). To further investigate a possible link between FGF7/FGFR2‐JNK axis and GSK3β‐mediated JunB phosphorylation, T47D cells were pretreated with SP600125 in the presence of E2 and P4 and then stimulated with FGF7. It was found that inhibition of JNK activity resulted in decreased GSK3β S9 and increased JunB T255 phosphorylation (Fig. 5D,E). This suggests that FGF7‐induced JunB accumulation is associated with JNK‐dependent GSK3β inactivation.

To assess the importance of JunB as a critical downstream effector of FGF7‐triggered signalling in hormone‐dependent BCa growth, JunB expression was silenced with specific shRNA (Fig. 5F). The growth of T47D and T47D shJunB_2 cells was analysed in 3D matrigel, MTT and colony formation assays in the presence of E2 and/or P4 ± FGF7. The results showed that loss of JunB expression was associated with a worse response to E2 stimulation, and more importantly, it abrogated the effects of FGF7 exerted on cell growth, in the presence of E2 and P4 (Fig. 5G–I and Fig. S8C). This finding reveals a hitherto unknown mechanistic link between FGFR2 signalling, JunB and BCa cell response to steroid hormones.

A prognostic value of *JUNB* expression in relation to the menopausal statuses was further investigated in the *in silico* analyses data and found to follow (only in METABRIC data set) the tendency observed for FGFR2, that is, a trend for an association of *JUNB*high status with longer OS found in the postmenopausal group was lost in premenopausal patients (Fig. 5J). Summing up *in vitro* and clinical analyses, we found that FGFR2➔JunB axis promotes ER+/PR+ BCa progression in premenopausal patients.

## 4 Discussion

Our study demonstrates for the first time that the role of FGFR2 in ER+ BCa is dependent on the hormonal background of the tumour and relies on the involvement of the FGF7/FGFR2➔JunB axis in the regulation of PR modulatory effects on ER‐associated cellular events. The impact of the hormonal context on the biological outcome of this newly identified mechanism is supported by the results of clinical analyses demonstrating that the prognostic value of the *FGFR2* and *JUNB* expression in ER+/PR+ BCa depends on the menopausal status of the patient.

Functional reports linking FGFR2 signalling with the activity of steroid hormone receptors have brought new insights into the TME‐induced regulation of both ER and PR, that influence BCa progression and response to anti‐ER drugs [14, 49]. However, as indicated by previous studies, including our own data [20], the role of FGFR2 in luminal BCa is complex and context‐dependent. This seems to reflect not only interactions of FGFR2‐triggered signalling separately with ER or PR, but also an impact of FGFR2 on the ER‐PR crosstalk, hence the dependence of the functional outcome of its activity on the hormonal milieu of the tumour.

Results of our *in vitro* studies on the role of FGFR2 in ER+PR+ BCa growth and response to tamoxifen in relation to the hormonal background showed that FGF7/FGFR2 signalling promoted proliferation of BCa cells and resistance to tamoxifen. This is in agreement with previously published data on the involvement of FGFR2 in regulation of ER, PR and BCa cell response to anti‐ER drugs [18, 19]. Additionally, simultaneous application of E2 and P4 (hormonal milieu typical for premenopausal patients) showed that FGFR2 activity counteracted the effects of P4 on BCa cell growth and response to tamoxifen. On the molecular level, FGF7/FGFR2 signalling triggered phosphorylation and turnover of both ER and PR, specifically in the presence of oestradiol and progesterone. This is in line with documented evidence of FGFR2‐induced phosphorylation of ER and PR, which leading to increased transcriptional activity and rapid degradation of both receptors, contributes to development of hormone‐independent BCa growth and resistance anti‐ER treatment [16, 17, 18, 19, 50, 51, 52, 53]. We also demonstrated here that JunB (a member of the AP‐1 family of transcription factors) was involved in FGF7‐triggered abolishment of inhibitory effects of P4 on ER‐dependent BCa growth. Furthermore, JNK pathway (known to activate members of the Jun family [54]) was identified as a major mediator of FGF7/FGFR2 signalling in the presence of steroid hormones. Stimulation of BCa cells with FGF7 increased protein level of JunB in a JNK‐dependent manner. Further analysis revealed that GSK3β kinase, a well‐known upstream modulator of JunB [44, 45] was involved in FGF7‐promoted increase of JunB expression. While a direct interdependence between JNK and GSK3β remains controversial [55, 56, 57, 58], our results identify GSK3β as a downstream effector of the FGFR2‐JNK axis implicated in the regulation of JunB protein level. The precise underlying molecular mechanisms of this regulation are still not revealed, but recent data suggest that the activity of Itch and FBXW7 E3 ubiquitin ligases, mediating posttranslational modifications, for example, ubiquitination and neddylation, might affect JunB cellular levels [44, 59].

**Fig. 6:**
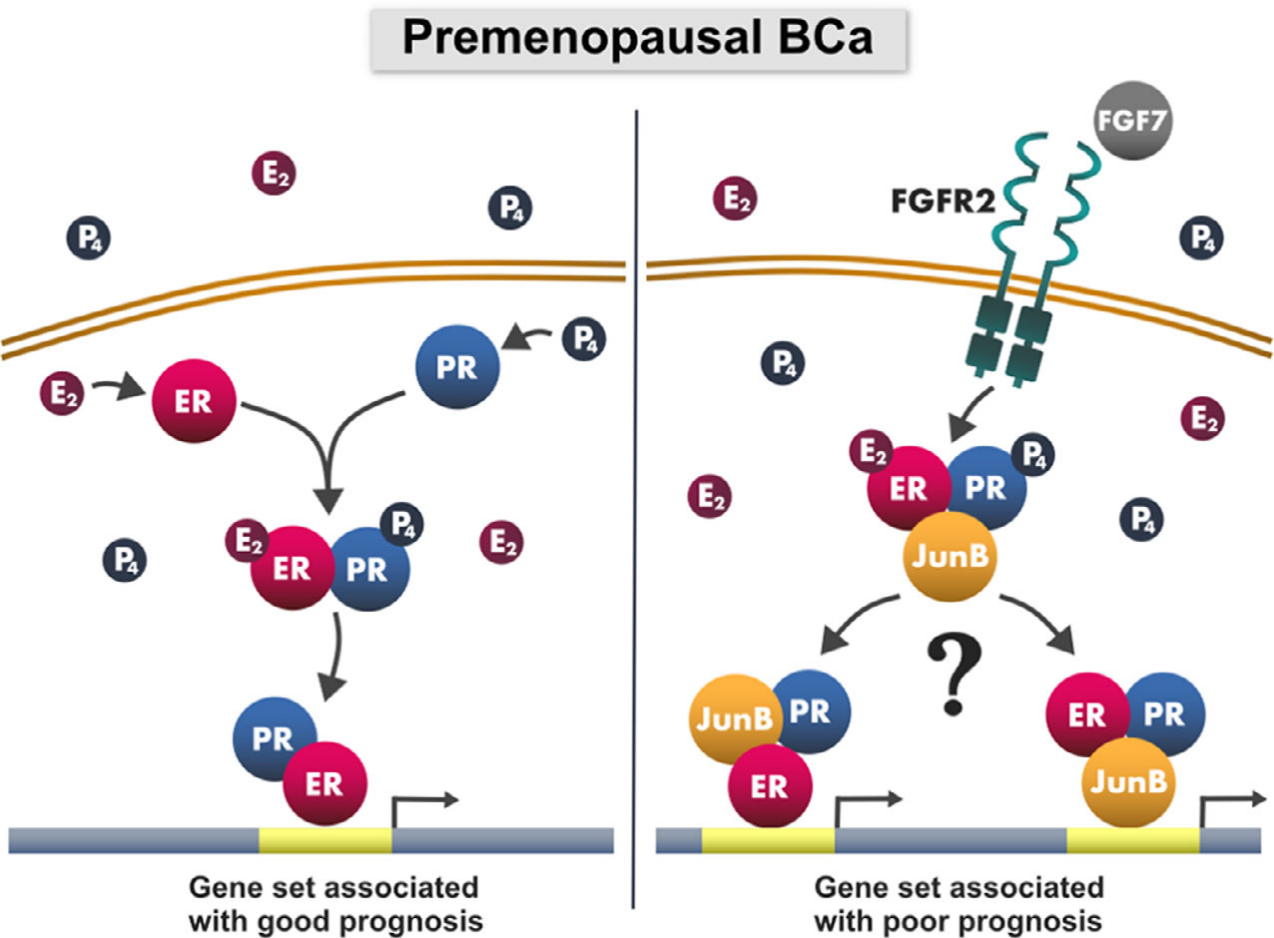
Proposed mechanism of the involvement of JunB in FGF7/FGFR2 signalling in hormone‐dependent (premenopausal) breast cancer (BCa). In the presence of both oestrogen (E2) and progesterone (P4) ER and PR form a direct complex which regulates the expression of a gene set associated with good prognosis (left panel). FGF7/FGFR2‐triggered signalling changes the composition of the steroid hormones‐induced complex, which leads to expression of a gene set associated with poor prognosis (right panel).

Giulianelli et al. demonstrated that FGF2 promoted tumour growth through an increase of interactions between ER and PR, and to date, this is the only study that recognises a significance of growth factors‐induced signalling for the functional relationship between the two receptors [60]. Our results indicating that FGF7/FGFR2 additionally enhanced steroid hormone‐induced interactions between ER and PR add another dimension to the crosstalk between luminal BCa and its setting. Interestingly, the pattern of expression of P4‐modulated ER‐dependent genes upon FGF7 stimulation was similar to that induced by treatment with E2 alone. These results contradict the previously published data, where in the presence of E2 (imitating postmenopausal conditions), FGF10/FGFR2 signalling was found to reverse the activity of *ESR1* regulon, driving BCa towards basal‐like phenotype [35, 61]. These discrepancies might be explained by ligand specificity (FGF10 vs. FGF7), and more importantly, dependent on hormonal background applied in these studies (i.e. reflecting pre‐ vs. postmenopausal status). Our data suggest that FGF7‐dependent effects may not result entirely from the increased number of ER–PR complexes but also from the recruitment of additional coactivators/transcription factors, for example, JunB, affecting ER transcriptional activity [62, 63] (a proposed model of FGFR2 action is presented in Fig. 6).

Results of our clinical analyses supported the mechanistic studies indicating that in premenopausal, in contrast to postmenopausal ER+PR+ patients, FGFR2 tends to associate with a bad prognosis. This suggests that the role of FGFR2 in the steroid hormones‐driven environment is more complex and involves additional factors affecting patients' outcome. We are aware of the limitations of our analyses, that is, a low number of patients in the premenopausal group. These are inherent to this specific subgroup of patients, that is, the low frequency of BCa in young (premenopausal) women, which when stratified into FGFR2 positive and negative cases prevents reaching statistical significance. In addition, FGFR2 may not represent the dominant driver of BCa pathophysiology, and hence, the low statistical significance of analyses of heterogenous cohorts of patients was rather to be expected. However, as long as the findings are confirmed in the independent databases (what has been done in this study) the data do have clinical implications and may be important for the identification of a BCa subtype likely to respond to the targeted (anti‐FGFR) therapy.

Interestingly, FGFR2 (protein) level was found to positively correlate with *JUNB* expression specifically in the premenopausal subgroup, suggesting that JunB is be potentially involved in FGFR2‐dependent regulation of ER transcriptional activity. Further clinical analyses showed that prognostic significance of *JUNB* mRNA followed the tendency displayed by FGFR2, that is, a trend for the association of FGFR2 with good prognosis in postmenopausal cohort was lost in the premenopausal group.

In summary, our combined molecular and clinical analyses of ER+PR+ BCa revealed that the role of FGFR2 depends on the menopausal status of the patients. FGF7/FGFR2**–**JunB axis was shown to abrogate the modulatory effect of PR on BCa cells and promote progression of premenopausal tumours.

## 5 Conclusions

In this study, we showed that FGF7/FGFR2 signalling abrogates progesterone effects and response to tamoxifen in ER‐dependent BCa cells. At the molecular level, in the presence of steroid hormones, FGF7 regulates the formation of ER‐PR complex and transcriptional activity of ER, abrogating the effects of P4 on expression of the ER‐dependent genes. Clinical analyses revealed that the prognostic value of FGFR2 in ER+ BCa depends on the menopausal status of the patients. High expression of FGFR2 had a tendency to associate with bad prognosis of premenopausal BCa which was reverted in postmenopausal cohort. Additional Nanostring‐based RNA quantitative analysis of BCa samples identified a positive strong correlation between FGFR2 and *JUNB*, specifically in premenopausal patients. FGF7/FGFR2**–**JunB axis was proved to counteract a negative effect of progesterone on ER+ BCa cell growth. Taken together, this study highlights a need for further stratification of premenopausal patients to select those who may benefit from the combination of endocrine and FGFR‐targeting therapies.

## Conflict of interest

The authors declare no conflict of interest.

## Author contributions

Conceptualization: KM, KK, MB, RK, HMR and RS; Data curation: KM, KK, MB; Formal analysis: KM, KK, MB; Funding acquisition: KM and RS; Investigation: KM, KK, MB, DN, and BG‐B; Methodology: KM, KK, MB, MG‐A, KS, AJZ; Project administration: RS; Resources: KM, KK, MB, HMR and RS; Software: KM, KK, MB; Supervision: HMR and RS; Validation: KM, KK, MB; Visualisation: KM, KK, MB; Writing – original draft: KM, KK, MB and RS; Writing – review & editing: KM, KK, MB, HMR and RS. All authors have read the journal's authorship agreement.

### Peer Review

The peer review history for this article is available at https://publons.com/publon/10.1002/1878‐0261.13274.

## Supporting information


**Fig. S1.** FGF7/FGFR2 abrogates the negative effect of P4 on T47D and CAMA‐1 cells growth.

**Fig. S2.** FGF7 abrogates the negative effect of P4 on E2‐dependent CAMA‐1 cells growth.

**Fig. S3.** FGF7/FGFR2 abrogates the negative effect of P4 on E2‐dependent T47D cells growth.

**Fig. S4.** FGF7/FGFR2 signalling regulates phosphorylation and expression level of PR and ER.

**Fig. S5.** FGF7/FGFR2 signalling affects P4 effect on ER‐PR complex formation.

**Fig. S6.** Expression of FGFR2 does not differ between pre‐ and postmenopausal breast cancer (BCa) patients.

**Fig. S7.** Involvement of PI3K/AKT and Src pathways in FGF7‐regulated hormone‐dependent breast cancer (BCa) cell growth.

**Fig. S8.** Activity of GSK3β is involved in regulation of JunB expression.

**Table S1.** Antibodies used in the study.
**Table S2.** Pathological and clinical characteristics of the study group and within subgroups dependent on menopausal status (data available for 231/246, 93.9% patients) and FGFR2 protein level.
**Table S3.** Analysis of correlation between FGFR2 protein levels and mRNA levels of *in vitro* specified biomarkers of ER activity (based on RT2 Oestrogen Receptor Signalling PCR Array) in clinical material.

**Table S4.** Summary of mRNA levels of *in vitro* specified biomarkers of ER activity (based on RT2 Oestrogen Receptor Signalling PCR Array) in clinical material for the whole group and with analysis of differences between pre‐ and postmenopausal patients.

## Data Availability

The data that support the findings of this study are available from the corresponding authors upon reasonable request.
