# Supplementary figures and images for: FGF7/FGFR2–JunB signalling counteracts the effect of progesterone in luminal breast cancer
Source: Mol Oncol. 2022 Jul 4;16(15):2823–42. doi: 10.1002/1878-0261.13274 (PMC9348598; doi:10.1002/1878-0261.13274)

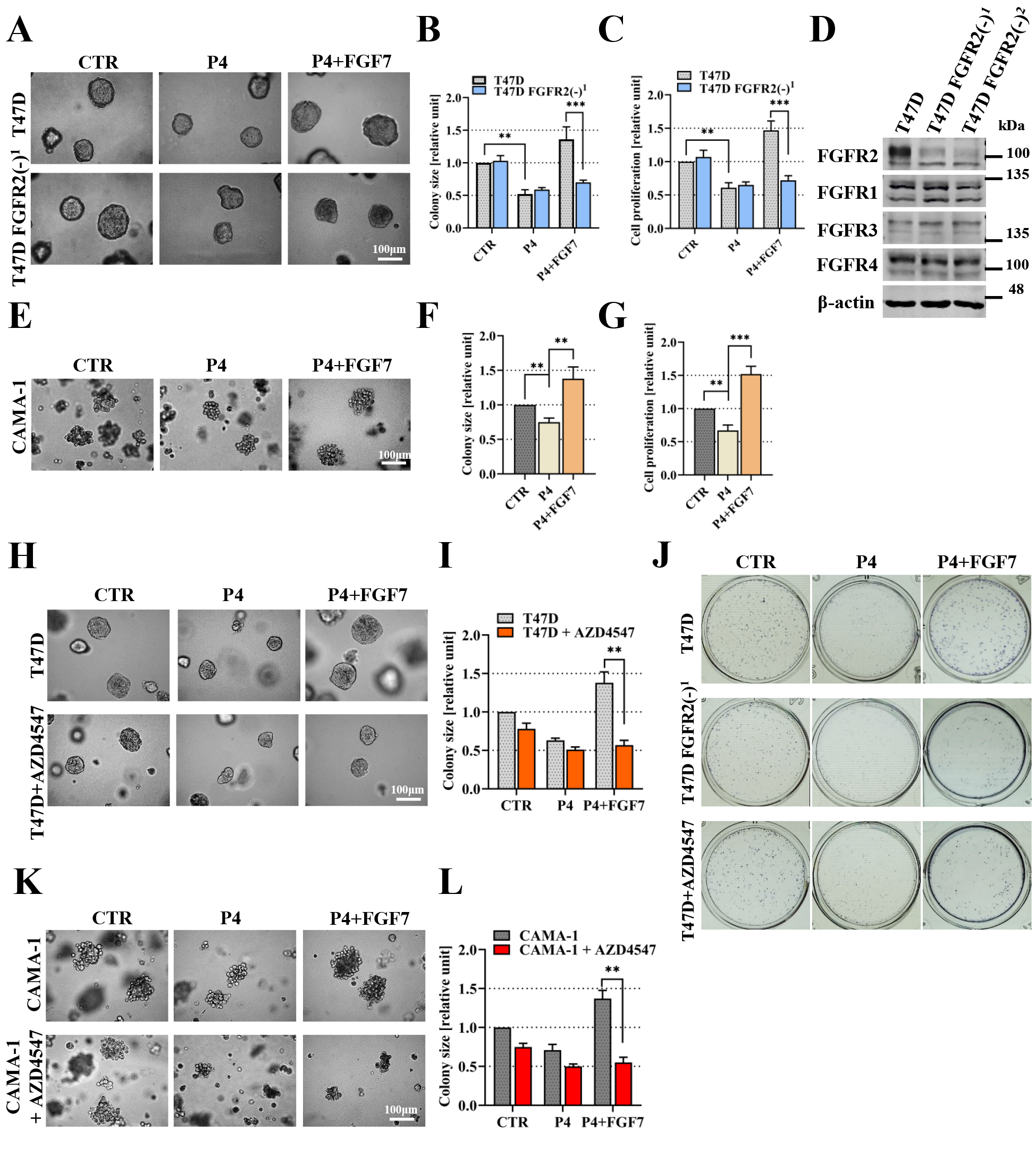

Supplement: Supplementary file 1 — Fig. S1. FGF7/FGFR2 abrogates the negative effect of P4 on T47D and CAMA‐1 cells growth. [file MOL2-16-2823-s006.tif]

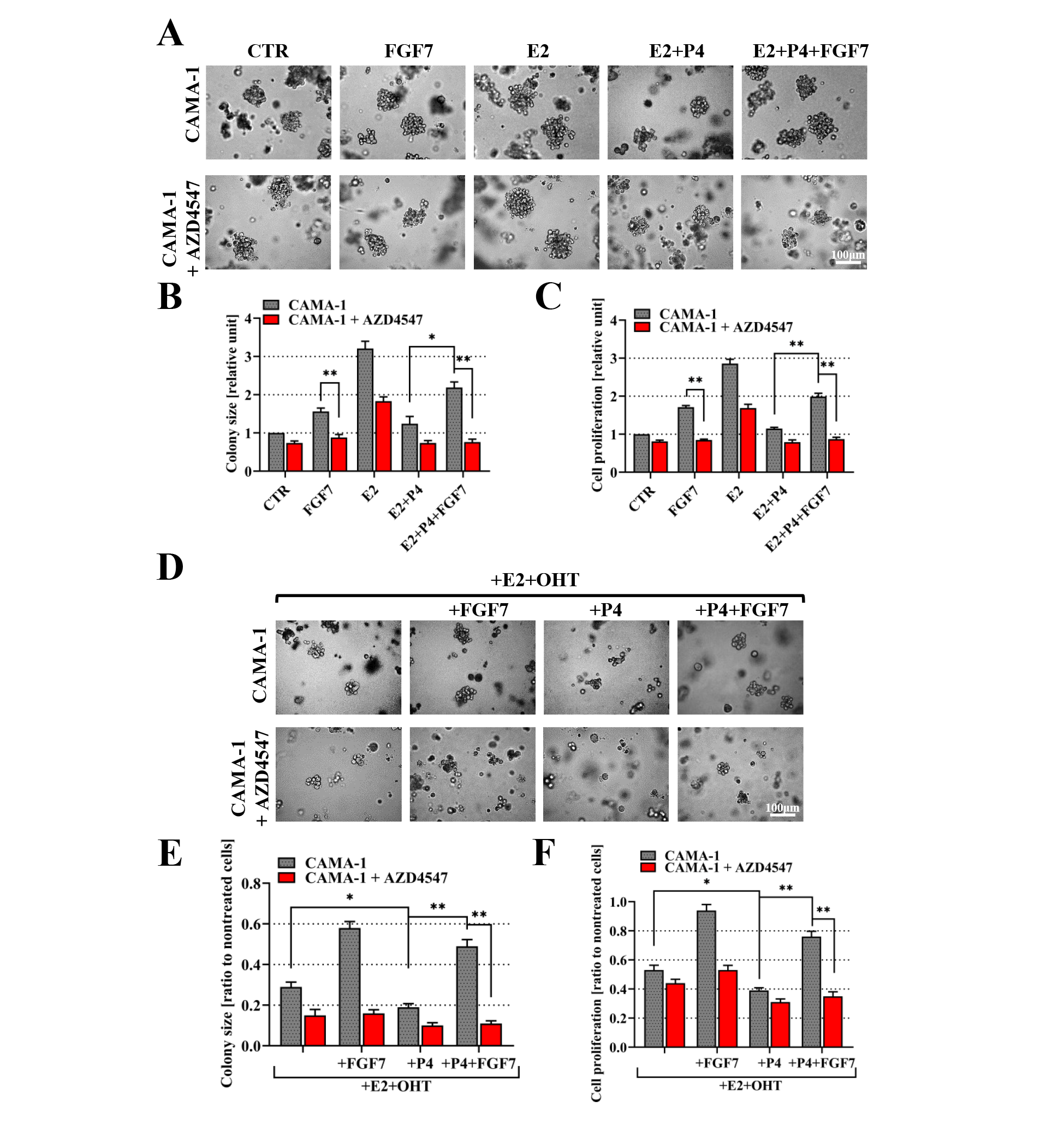

Supplement: Supplementary file 2 — Fig. S2. FGF7 abrogates the negative effect of P4 on E2‐dependent CAMA‐1 cells growth. [file MOL2-16-2823-s007.tif]

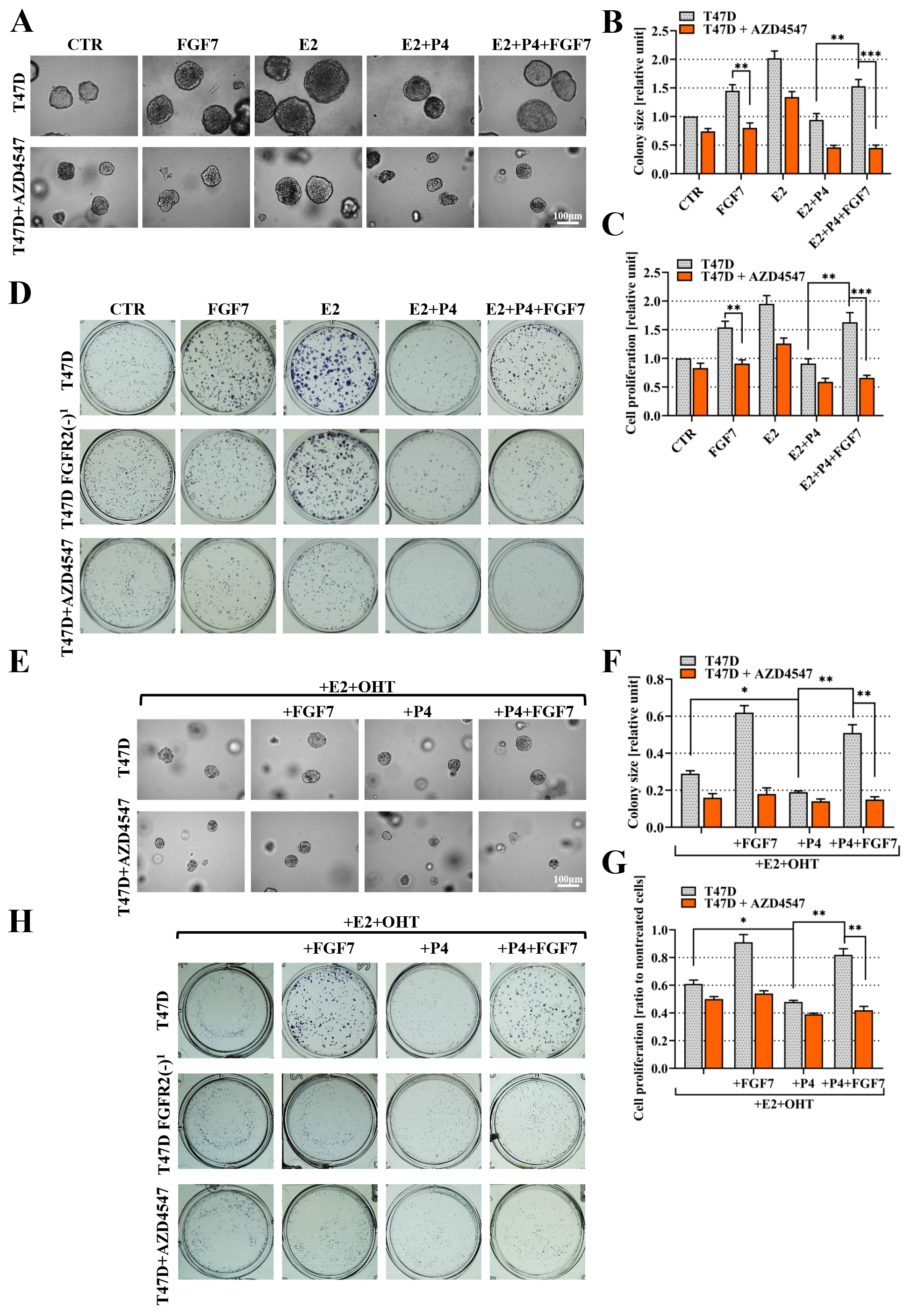

Supplement: Supplementary file 3 — Fig. S3. FGF7/FGFR2 abrogates the negative effect of P4 on E2‐dependent T47D cells growth. [file MOL2-16-2823-s002.tif]

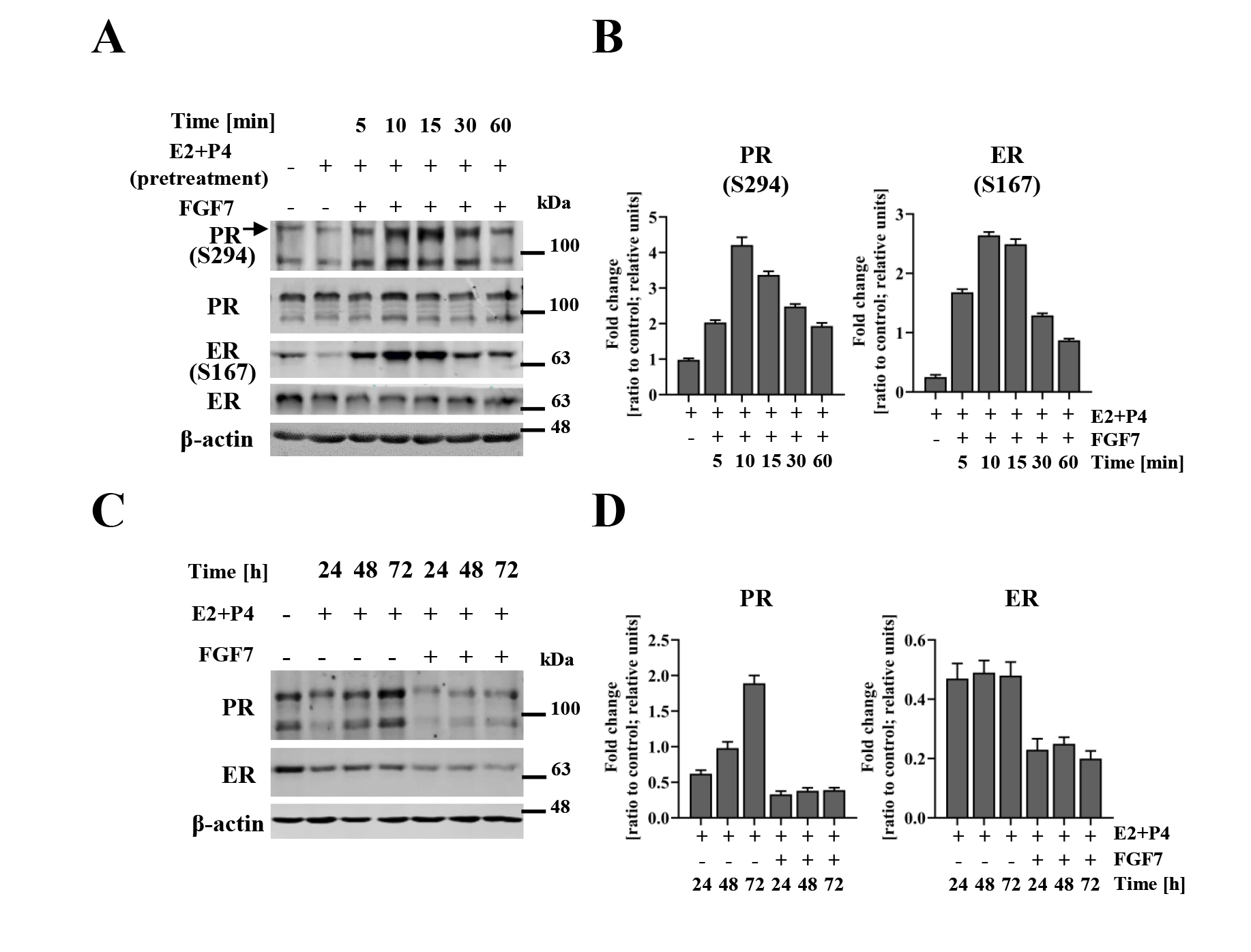

Supplement: Supplementary file 4 — Fig. S4. FGF7/FGFR2 signalling regulates phosphorylation and expression level of PR and ER. [file MOL2-16-2823-s004.tif]

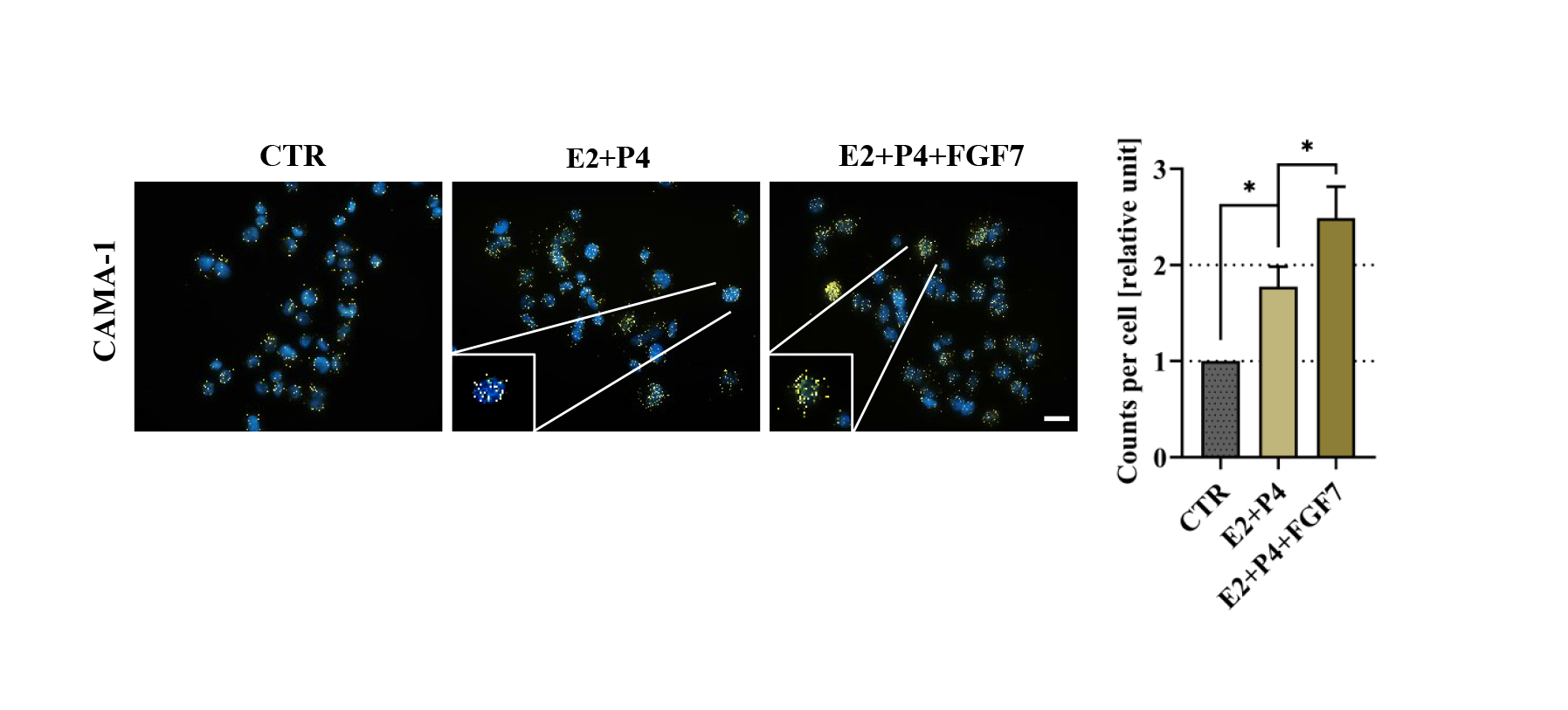

Supplement: Supplementary file 5 — Fig. S5. FGF7/FGFR2 signalling affects P4 effect on ER‐PR complex formation. [file MOL2-16-2823-s001.tif]

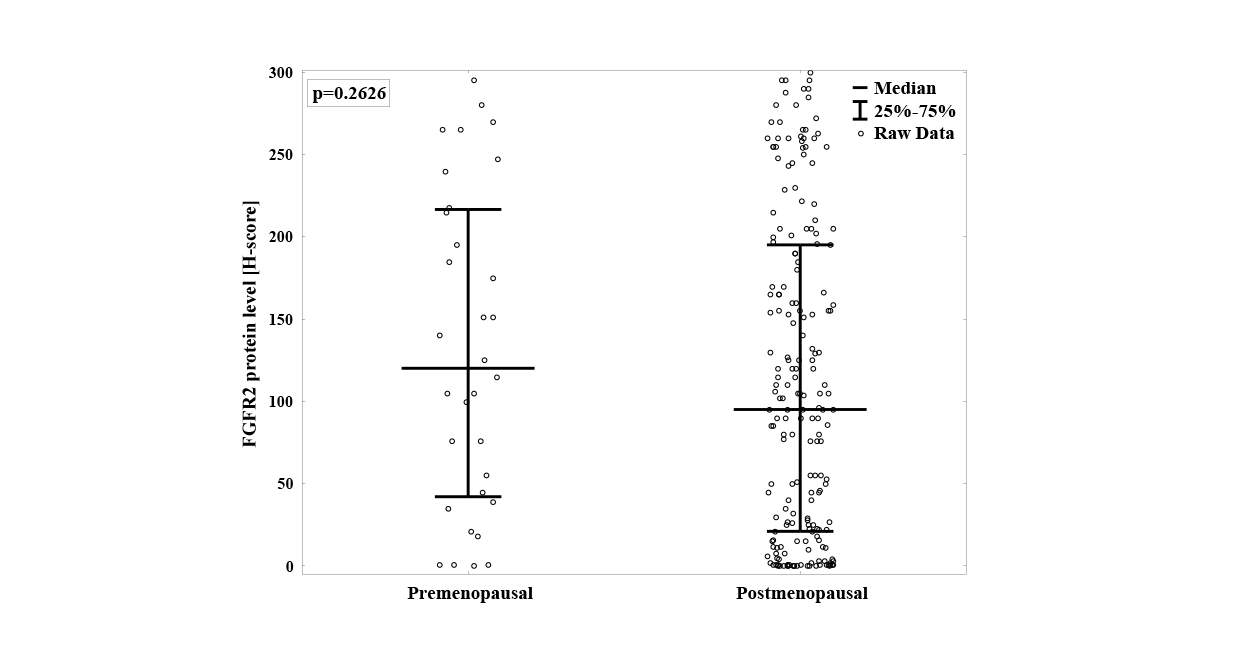

Supplement: Supplementary file 6 — Fig. S6. Expression of FGFR2 does not differ between pre‐ and postmenopausal breast cancer (BCa) patients. [file MOL2-16-2823-s010.tif]

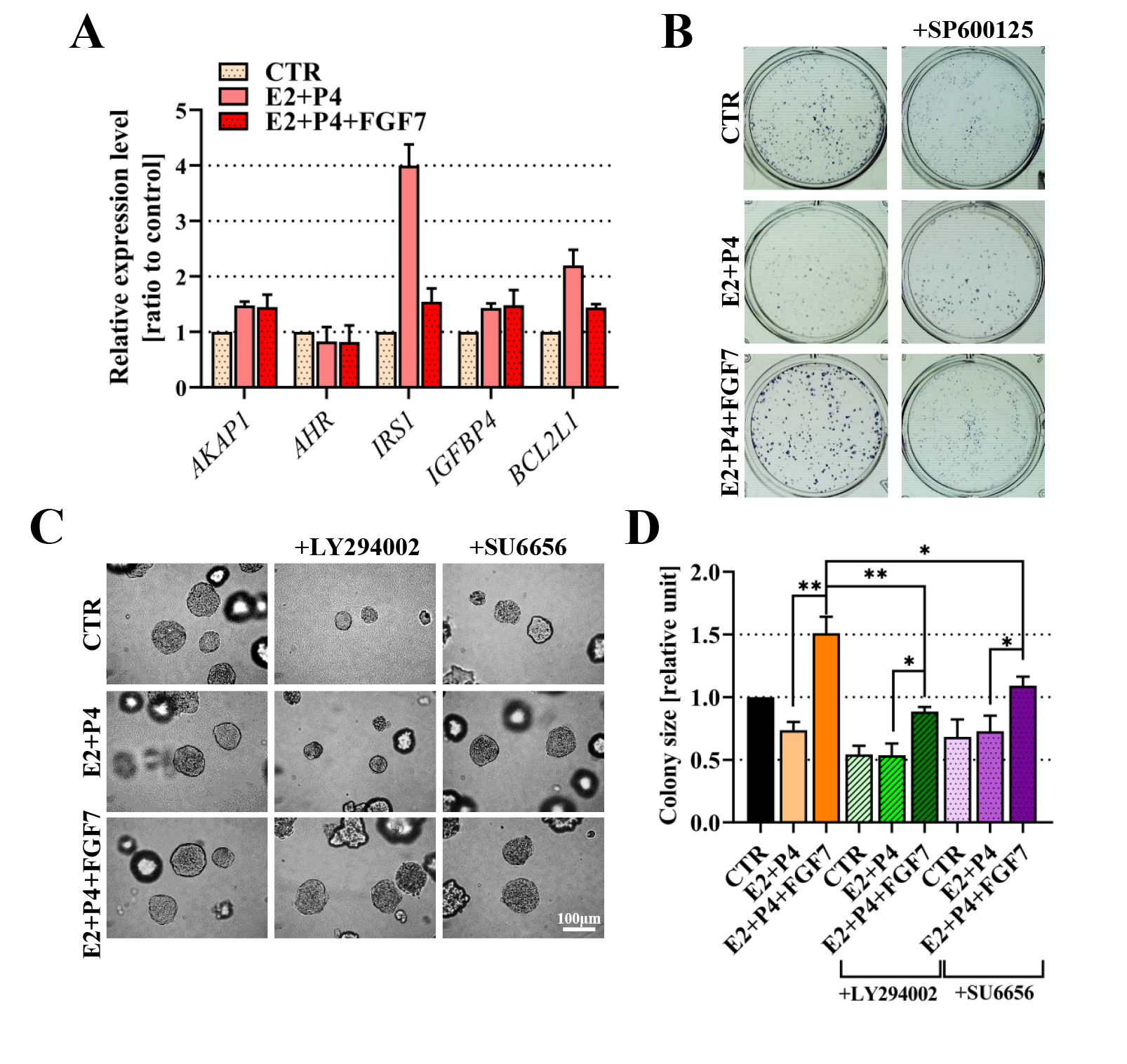

Supplement: Supplementary file 7 — Fig. S7. Involvement of PI3K/AKT and Src pathways in FGF7‐regulated hormone‐dependent breast cancer (BCa) cell growth. [file MOL2-16-2823-s005.tif]

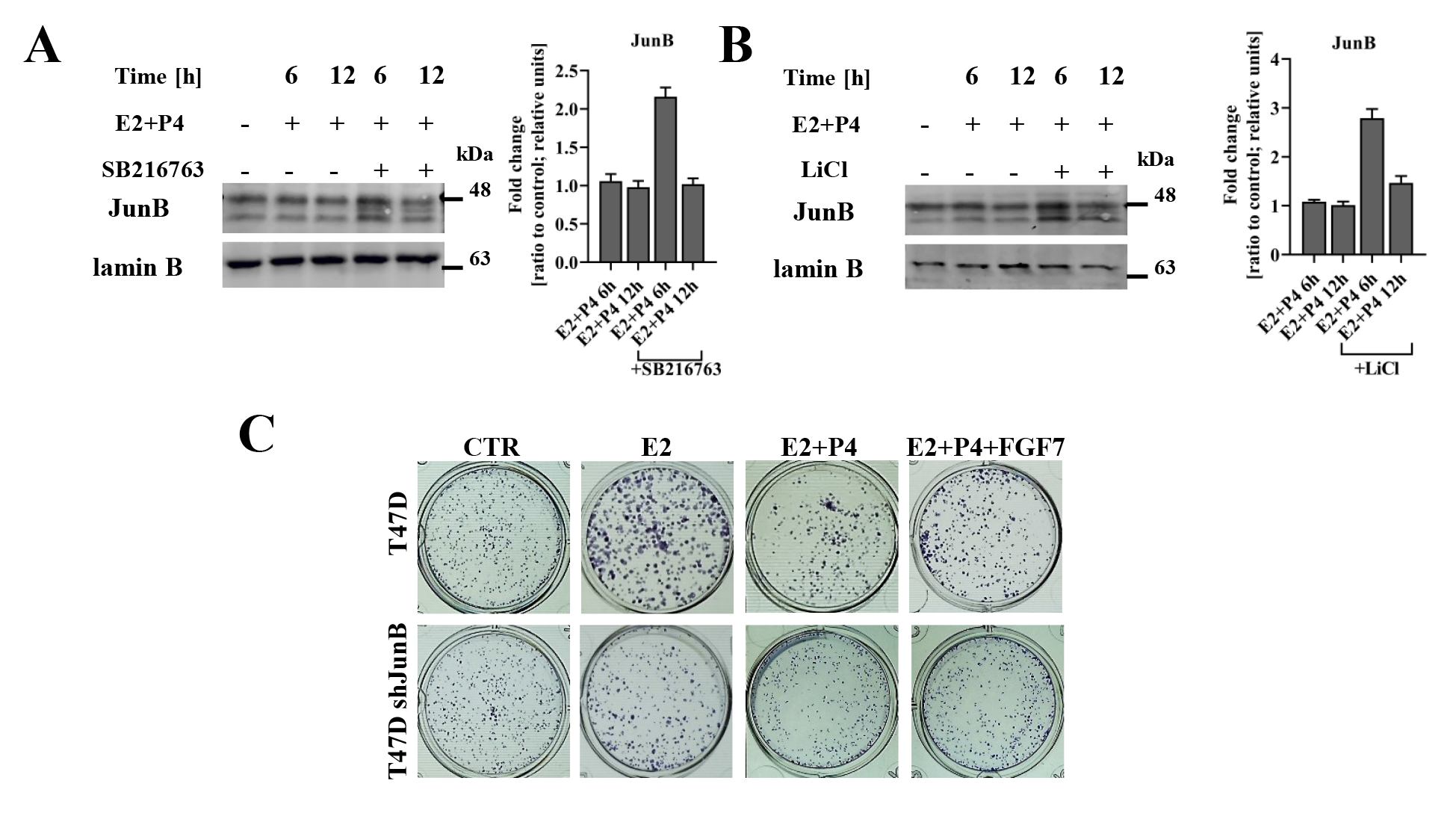

Supplement: Supplementary file 8 — Fig. S8. Activity of GSK3β is involved in regulation of JunB expression. [file MOL2-16-2823-s003.tif]
