# Supplementary material for: FGF7/FGFR2–JunB signalling counteracts the effect of progesterone in luminal breast cancer
Source: Mol Oncol. 2022 Jul 4;16(15):2823–42. doi: 10.1002/1878-0261.13274 (PMC9348598; doi:10.1002/1878-0261.13274)
Supplement: Supplementary file 9 — Table S1. Antibodies used in the study. Table S2. Pathological and clinical characteristics of the study group and within subgroups dependent on menopausal status (data available for 231/246, 93.9% patients) and FGFR2 protein level. Table S3. Analysis of correlation between FGFR2 protein levels and mRNA levels of in vitro specified biomarkers of ER activity (based on RT2 Oestrogen Receptor Signalling PCR Array) in clinical material. [file MOL2-16-2823-s009.docx]

**Supplementary Table 1.**

Antibodies used in the study.

| Antibodies | Source | Application |
| --- | --- | --- |
| Mouse monoclonal anti-β-actin | Sigma-Aldrich; clone AC-74 | WB |
| Rabbit monoclonal anti-ERα | Thermo Fisher; clone SP1 | WB, PLA |
| Mouse monoclonal anti-ERα | Thermo Fisher; clone 1D5 | IP |
| Rabbit monoclonal anti-pER (S167) | Cell Signaling; #64508 | WB |
| Rabbit monoclonal anti-GSK3β | Cell Signaling; #12456 | WB |
| Rabbit monoclonal anti-pGSK3β (S9) | Cell Signaling; #5558 | WB |
| Rabbit polyclonal anti-pJNK (T183/Y185) | Cell Signaling; #9251 | WB |
| Rabbit monoclonal anti-JunB | Cell Signaling; #3753 | WB |
| Rabbit monoclonal anti-JunD | Cell Signaling; #5000 | WB |
| Rabbit monoclonal anti-c-Jun | Cell Signaling; #9165 | WB |
| Rabbit polyclonal anti-pJunB (T255) | Thermo Fisher; #PA5-105510 | WB |
| Mouse monoclonal anti-PRA/B | Thermo Fisher; clone hPRa7 | PLA |
| Rabbit monoclonal anti-PRA/B | Cell Signaling; #8757 | WB |
| Mouse monoclonal anti-pPR (S294) | Thermo Fisher; #MA1-414 | WB |
| Donkey anti-mouse AlexaFluor® 790 | Jackson ImmunoResearch | WB |
| Goat anti-rabbit AlexaFluor® 680 | Jackson ImmunoResearch | WB |

WB – Western blotting, PLA – proximity ligation assay, IP - immunoprecipitation

**Supplementary Table 2**.

Pathological and clinical characteristics of the study group and within subgroups dependent on menopausal status (data available for 231/246, 93.9% patients) and FGFR2 protein level. Nominal variables are presented as raw values followed by percentages of the eligible groups, and continuous variables are presented as medians and interquartile ranges in brackets; DCIS – ductal carcinoma *in situ*, HT – hormonotherapy, CHTH – chemotherapy, RTH – radiotherapy;

* Kruskal-Wallis ANOVA test, ** log-rank test, *** Pearson's chi-squared test.

| Variable | Whole group (ER+PR+)  n=246 (100.0) | FGFR2low/  Premenopausal  n=6/231 (2.6) | FGFR2high/  Premenopausal  n=26/231 (11.3) | FGFR2high/  Postmenopausal  n=138/231 (59.7) | FGFR2low/  Postmenopausal  n=61/231 (26.4) | p |
| --- | --- | --- | --- | --- | --- | --- |
| Age (years) | 63.6  (52.7-70.8) | 38.1  (37.0-42.5) | 41.2  (35.9-42.6) | 66.3  (59.3-72.7) | 65.2  (59.3-75.9) | <0.001^*^ |
| Grade  1  2  3 | 31 (12.6)  168 (69.9)  42 (17.5) | 0 (0.0)  3 (50.0)  3 (50.0) | 2 (7.7)  20 (76.9)  4 (15.4) | 20 (14.5)  98 (71.0)  20 (14.5) | 6 (9.8)  41 (67.2)  14 (22.9) | 0.237^***^ |
| Ki67 % | 12.0  (5.0-25.0) | 25.0  (25.0-25.0) | 10.0  (5.0-20.0) | 12.0  (5.0-26.5) | 12.0  (5.0-14.0) | 0.530^*^ |
| HER2 amplification positivity | 20.0 (8.1) | 0 (0.0) | 3 (11.5) | 12 (8.7) | 3 (4.9) | 0.599^***^ |
| Tumour size (mm) | 20.0  (15.0-25.0) | 19.5  (13.0-30.0) | 18.0  (12.0-25.0) | 20.0  (15.5-25.0) | 19.0  (15.0-25.0) | 0.828^*^ |
| T feature  pT1  pT2  pT3-4 | 115 (57.8)  78 (39.2)  6 (3.0) | 4 (66.7)  2 (33.3)  0 (0.0) | 11 (55.0)  9 (45.0)  0 (0.0) | 62 (57.4)  42 (38.9)  4 (3.7) | 35 (58.3)  23 (38.3)  2 (3.3) | 0.971^***^ |
| Metastases present | 77 (32.0) | 4 (66.7) | 12 (46.1) | 40 (29.6) | 9 (32.2) | 0.122^***^ |
| N feature  pN0  pN1  pN2-3 | 164 (68.0)  55 (22.8)  22 (9.1) | 3 (57.6)  2 (30.3)  1 (12.1) | 14 (53.8)  5 (19.2)  7 (26.9) | 96 (71.1)  31 (23.0)  8 (5.9) | 42 (71.2)  14 (23.7)  3 (5.1) | 0.023^***^ |
| Staging  Very early (IA)  Early  (IB-IIIA)  Advanced (IIIB-IV) | 101 (41.9)  127 (52.7)  13 (5.4) | 1 (16.7)  5 (83.3)  0 (0.0) | 10 (38.5)  14 (53.8)  2 (7.7) | 61 (44.9)  65 (47.8)  10 (7.3) | 24 (40.7)  35 (59.3)  0 (0.0) | 0.218^***^ |
| Multifocality | 30 (15.0) | 1 (16.7) | 7 (35.0) | 12 (11.1) | 10 (16.7) | 0.058^***^ |
| DCIS present | 70 (28.3) | 3 (50.0) | 15 (57.7) | 30 (21.7) | 16 (26.2) | 0.001^***^ |
| HT | 174 (86.6) | 2 (66.7) | 22 (88.0) | 97 (85.1) | 42 (89.4) | 0.671^***^ |
| Adjuvant CHTH | 87 (56.9) | 2 (66.7) | 22 (88.0) | 45 (49.5) | 11 (50.0) | 0.006^***^ |
| Adjuvant RTH | 110 (67.9) | 2 (66.7) | 20 (89.7) | 62 (64.6) | 16 (61.5) | 0.189^***^ |
| Progression/ Relapse | 12 (5.8) | 0 (0.0) | 1 (4.8) | 11 (9.6) | 14 (26.4) | 0.080^**^ |
| Disease-free survival (years) | 4.2 (2.9-6.6) | 6.7 (3.9-7.1) | 3.9 (2.6-5.0) | 4.0 (2.8-6.5) | 4.4 (2.9-6.7) |  |
| Deaths | 25 (10.1) | 0 (0.0) | 1 (3.8) | 10 (7.3) | 14 (22.9) | 0.041^**^ |
| Overall survival (years) | 4.7 (3.0-6.7) | 6.7 (6.4-7.0) | 4.3 (3.4-5.9) | 4.1 (2.8-6.8) | 6.4 (3.1-6.8) |  |

**Supplementary Table 3.**

Analysis of correlation between FGFR2 protein levels and mRNA levels of *in vitro* specified biomarkers of ER activity (based on RT2 Estrogen Receptor Signalling PCR Array) in clinical material. Spearman correlation coefficients with raw p-values and Benjamini-Hochberg (BH) corrected p-values for whole group (n=246) are presented. Genes are selected according to significance level of correlations.

| Correlation between  FGFR2 protein [H-score] levels with  mRNA [log2] levels of: | Correlation coefficient (R) | Raw p-value | BH-corrected  p-value |
| --- | --- | --- | --- |
| *BCL2L1* | 0.25 | <0.0001 | 0.0017 |
| *S100A6* | 0.22 | 0.0005 | 0.0053 |
| *IRS1* | 0.2 | 0.0019 | 0.0113 |
| *AHR* | 0.19 | 0.0024 | 0.0113 |
| *JUNB* | 0.19 | 0.0027 | 0.0113 |
| *MMP9* | 0.17 | 0.0067 | 0.0196 |
| *EFNA5* | 0.17 | 0.0072 | 0.0196 |
| *BRCA1* | 0.17 | 0.0074 | 0.0196 |
| *CCL2* | 0.15 | 0.0182 | 0.0426 |
| *CKB* | 0.14 | 0.0258 | 0.0514 |
| *LGALS1* | 0.14 | 0.0270 | 0.0514 |
| *NCOA3* | 0.13 | 0.0443 | 0.0774 |
| *NR3C1* | 0.11 | 0.0815 | 0.1265 |
| *TGFB3* | 0.11 | 0.0843 | 0.1265 |
| *LPL* | 0.11 | 0.0993 | 0.1390 |
| *CITED2* | 0.10 | 0.1325 | 0.1739 |
| *ESR1* | 0.07 | 0.2837 | 0.3504 |
| *EBAG9* | -0.06 | 0.3302 | 0.3853 |
| *AKAP1* | 0.05 | 0.4027 | 0.4451 |
| *BMP4* | 0.04 | 0.4799 | 0.5038 |
| *BMP7* | -0.01 | 0.8290 | 0.8290 |

**Supplementary Table 4.**

Summary of mRNA levels of *in vitro* specified biomarkers of ER activity (based on RT2 Estrogen Receptor Signalling PCR Array) in clinical material for the whole group and with analysis of differences between pre- and postmenopausal patients. Raw p-values from Mann–Whitney U test followed by Benjamini-Hochberg (BH) corrected p-values are presented. Genes are selected according to significance level of differences.

| Gene | Median (IQR) expression [log2] whole group | Median (IQR) expression [log2] premenopausal | Median (IQR) expression [log2] postmenopausal | Raw  p-value | BH-corrected p-value |
| --- | --- | --- | --- | --- | --- |
| *ESR1* | 11.74 (10.98-12.57) | 10.82 (10.12-11.22) | 11.99 (11.2-12.73) | <0.0001 | <0.0001 |
| *CKB* | 7.64 (6.75-8.38) | 7.76 (7.13-9.09) | 7.59 (6.68-8.35) | 0.0379 | 0.2726 |
| *IRS1* | 8.00 (7.27-8.51) | 8.19 (7.71-8.76) | 7.92 (7.14-8.4) | 0.0389 | 0.2726 |
| *CITED2* | 10.31 (9.81-10.9) | 9.97 (9.61-10.68) | 10.36 (9.84-10.93) | 0.0779 | 0.4092 |
| *TGFB3* | 9.28 (8.59-9.86) | 9.37 (8.78-9.86) | 9.18 (8.53-9.82) | 0.3015 | 0.7682 |
| *EBAG9* | 9.46 (9.09-9.88) | 9.39 (9.05-9.86) | 9.47 (9.11-9.88) | 0.3178 | 0.7682 |
| *NR3C1* | 8.32 (7.82-8.65) | 8.36 (8.14-8.59) | 8.28 (7.78-8.63) | 0.3276 | 0.7682 |
| *BMP4* | 7.36 (6.61-8.03) | 7.19 (6.57-7.84) | 7.34 (6.59-8.1) | 0.3754 | 0.7682 |
| *LPL* | 7.37 (6.55-8.07) | 7.36 (6.67-8.44) | 7.33 (6.48-7.95) | 0.3902 | 0.7682 |
| *JUNB* | 11.58 (11.01-12.22) | 11.43 (10.81-11.95) | 11.55 (10.99-12.15) | 0.4175 | 0.7682 |
| *AKAP1* | 7.99 (7.6-8.42) | 8.02 (7.66-8.58) | 7.98 (7.6-8.41) | 0.424 | 0.7682 |
| *LGALS1* | 12.88 (12.39-13.28) | 12.93 (12.55-13.32) | 12.86 (12.29-13.28) | 0.4544 | 0.7682 |
| *MMP9* | 8.20 (7.1-9.41) | 8.45 (6.85-10.12) | 8.05 (7.1-9.29) | 0.5039 | 0.7682 |
| *BMP7* | 5.61 (4.77-6.38) | 5.78 (5.09-6.34) | 5.52 (4.56-6.39) | 0.5121 | 0.7682 |
| *S100A6* | 12.02 (11.52-12.48) | 11.9 (11.51-12.36) | 11.98 (11.48-12.48) | 0.621 | 0.8258 |
| *NCOA3* | 8.91 (8.65-9.33) | 8.9 (8.65-9.2) | 8.9 (8.63-9.39) | 0.6535 | 0.8258 |
| *AHR* | 10.35 (9.92-10.76) | 10.39 (9.83-10.86) | 10.31 (9.88-10.69) | 0.6784 | 0.8258 |
| *BCL2L1* | 9.85 (9.51-10.2) | 9.78 (9.52-10.13) | 9.87 (9.51-10.19) | 0.7078 | 0.8258 |
| *CCL2* | 10.02 (9.34-10.67) | 9.86 (9.21-11.24) | 9.99 (9.42-10.55) | 0.7593 | 0.8392 |
| *EFNA5* | 6.55 (5.92-7.28) | 6.35 (5.95-7.06) | 6.49 (5.85-7.31) | 0.8923 | 0.9369 |
| *BRCA1* | 6.52 (6.12-7.08) | 6.6 (6.12-7) | 6.52 (6.12-7.12) | 0.9716 | 0.9716 |
