# Supplementary material for: FGF7/FGFR2–JunB signalling counteracts the effect of progesterone in luminal breast cancer
Source: Mol Oncol. 2022 Jul 4;16(15):2823–42. doi: 10.1002/1878-0261.13274 (PMC9348598; doi:10.1002/1878-0261.13274)
Supplement: Supplementary file 10 — Table S4. Summary of mRNA levels of in vitro specified biomarkers of ER activity (based on RT2 Oestrogen Receptor Signalling PCR Array) in clinical material for the whole group and with analysis of differences between pre‐ and postmenopausal patients. [file MOL2-16-2823-s008.docx]

**Supplementary legends**

**Supplementary Fig. S1 FGF7/FGFR2 abrogates the negative effect of P4 on T47D and CAMA-1 cells growth. (A-B)** T47D and T47D FGFR2(-)^1^ cells were cultured in 3D Matrigel for 14 days with P4 (progesterone, 100 nM) ± FGF7 (50 ng/ml). Representative images were taken (scale bar: 100 µm) **(A)** and colony size (relative to CTR/non-treated wild-type cells) **(B)** was analysed using ImageJ software. **(C)** The effect of P4 ± FGF7 on proliferation of T47D and T47D FGFR2(-)^1^ cells was analysed by MTT assay. Data for 3D Matrigel cultures and MTT assay are presented as means ± SD (n = 3), **p<0.005, ***p<0.001 by Student’s *t* test. **(D)** The specificity and efficiency of FGFR2 silencing with two shRNA constructs in T47D cells was confirmed by Western blotting. **(E-F)** CAMA-1 cells were cultured in 3D Matrigel for 14 days with P4 (100 nM) ± FGF7 (50 ng/ml). Representative images were taken **(E)** and colony size (relative to CTR/non-treated cells) **(F)** was analysed using ImageJ software. **(G)** The effect of P4 ± FGF7 treatment on proliferation of CAMA-1 cells was analysed by MTT assay. Data for 3D Matrigel cultures and MTT assay are presented as means ± SD (n = 3), **p<0.005, ***p<0.001 by Student’s *t* test. **(H-I)** T47D cells were cultured in 3D Matrigel for 14 days with P4 ± FGF7, ± AZD4547 (0.5 µM). Representative images were taken (scale bar: 100 µm) **(H)** and colony size (relative to CTR/non-treated conditions) **(I)** determined with ImageJ software. Data for 3D Matrigel cultures are presented as means ± SD (n = 3), **p<0.005 by Student’s *t* test. **(J)** Colony formation assay for T47D and T47D FGFR2(-)^1^ cells treated for 10 days with P4 ± FGF7, ± AZD4547. **(K-L)** CAMA-1 cells were cultured in 3D Matrigel for 14 days with P4 ± FGF7, ± AZD4547. Representative images were taken **(K)** and colony size (relative to CTR/non-treated conditions) **(L)** determined with ImageJ software. Data for 3D Matrigel cultures and MTT assay are presented as means ± SD (n = 3), **p<0.005, by Student’s *t* test.

**Supplementary Fig. S2 FGF7 abrogates the negative effect of P4 on E2-dependent CAMA-1 cells growth. (A-B)** CAMA-1 cells were cultured in 3D Matrigel for 14 days with E2 (oestrogen, 10 nM) and/or P4 (progesterone, 100 nM), and/or FGF7 (50 ng/ml) ± AZD4547 (0.5 µM). Representative images were taken (scale bar: 100 µm) **(A)** and colony size (relative to CTR/non treated conditions) **(B)** was analysed using ImageJ software. **(C)** The effect of E2 and/or P4, and/or FGF7 ± AZD4547 on CAMA-1 cells proliferation was analysed by MTT assay. Data for 3D Matrigel cultures and MTT assay are presented as means ± SD (n = 3), *p<0.05, **p<0.005 by Student’s *t* test. **(D-E)** CAMA-1 cells were cultured in 3D Matrigel for 14 days with E2 and/or P4, and/or FGF7 ± AZD4547 in the presence of OHT (4-hydroxytamoxifen, 1 µM). Representative images were taken (scale bar: 100 µm) **(D)** and average colony size (relative to CTR/non-treated conditions) **(E)** was established with ImageJ software. **(F)** The effect of E2 and/or P4, and/or FGF7 ± AZD4547 on CAMA-1 cells proliferation in the presence of OHT was analysed by MTT assay. Data for 3D Matrigel cultures and MTT assay are presented as means ± SD (n = 3), *p<0.05, **p<0.005 by Student’s *t* test.

**Supplementary Fig. S3 FGF7/FGFR2 abrogates the negative effect of P4 on E2-dependent T47D cells growth. (A-B)** T47D cells were cultured in 3D Matrigel for 14 days with E2 (oestrogen, 10 nM) and/or P4 (progesterone, 100 nM), and/or FGF7 (50 ng/ml) ± AZD4547 (0.5 µM). Representative images were taken (scale bar: 100 µm) **(A)** and colony size (relative to CTR/non-treated conditions) **(B)** was analysed using ImageJ software. **(C)** The effect of E2 and/or P4, and/or FGF7 ± AZD4547 treatment on proliferation of T47D cells was analysed by MTT assay. Data for 3D Matrigel cultures and MTT assay are presented as means ± SD (n = 3), **p<0.005, ***p<0.001 by Student’s *t* test. **(D)** Colony formation assay for T47D and T47D FGFR2(-)^1^ cells treated for 10 days with E2 and/or P4, and/or FGF7, ± AZD4547. **(E-F)** T47D cells were cultured in 3D Matrigel for 14 days with E2 and/or P4, and/or FGF7 ± AZD4547 in the presence of OHT (4-hydroxytamoxifen, 1 µM). Representative images were taken (scale bar: 100 µm) **(E)** and colony size (relative to CTR/non-treated conditions) **(F)** was determined with ImageJ software. **(G)** The effect of E2 and/or P4, and/or FGF7 ± AZD4547 in the presence of OHT on proliferation of T47D cells was analysed by MTT assay. Data for 3D Matrigel cultures and MTT assay are presented as means ± SD (n = 3), *p<0.05, **p<0.005 by Student’s *t* test. **(H)** Colony formation assay for T47D and T47D FGFR2(-)^1^ cells treated for 10 days with E2 and/or P4, and/or FGF7, ± AZD4547 in the presence of OHT.

**Supplementary Fig. S4 FGF7/FGFR2 signalling regulates phosphorylation and expression level of PR and ER. (A-B)** CAMA-1 cells were incubated for 24h in the presence of E2 (oestrogen, 10 nM) and P4 (progesterone, 100 nM) in serum-free phenol red-free medium and then treated with FGF7 (50 ng/ml) for 0-60 minutes. PR and ER phosphorylation was evaluated by Western blotting **(A)** and densitometry **(B)**. **(C-D)** CAMA-1 cells were treated with E2 (10 nM) and P4 (100 nM) or with the combination of both steroid hormones with FGF7 (50 ng/ml) for 0-72 hours. PR and ER expression level was evaluated by Western blotting **(C)** and densitometry **(D)**. Densitometry was done with Image StudioTMSoftware Ver 5.2, Odyssey CLx, data are presented as means ± SD (n=3).

**Supplementary Fig. S5 FGF7/FGFR2 signalling affects P4 effect on ER-PR complex formation.** The number of ER-PR complexes in CAMA-1 cells upon E2 (CTR; oestrogen, 10 nM), E2 with P4 (progesterone, 100 nM) or FGF7 (50 ng/ml) in the combination with E2 and P4 treatment was analysed using proximity ligation assay. Representative fluorescent microscopy images were taken (scale bar = 20 µm). Protein-protein interactions were quantified using ImageJ software and presented as a mean number of immunofluorescent dots per cell ± SD (n=3), *p<0.05 by Student’s *t* test.

**Supplementary Fig. S6 Expression of FGFR2 does not differ between pre- and postmenopausal breast cancer (BCa) patients.** FGFR2 protein level (H-score) was compared between premenopausal and postmenopausal patients, p=0.2626. P-value from Mann-Whitney U-test.

**Supplementary Fig. S7 Involvement of PI3K/AKT and Src pathways in FGF7-regulated hormone-dependent breast cancer (BCa) cell growth. (A)** A pilot study for selection of biomarkers of ER transcriptional activity. T47D cells were serum-starved in phenol red-free medium and treated with E2 (oestrogen, 10 nM) and P4 (progesterone, 100 nM), or FGF7 (50 ng/ml) with both steroid hormones for 6h. Relative expression of *AKAP1, AHR, IRS1, IGFBP4* and *BCL2L1* (selected from RT2 Profiler Estrogen Receptor Signaling PCR Array) was analysed by RT-qPCR. Each bar represents ratio to control mean ± SD (n=3). **(B)** Colony formation assay for T47D cells treated for 10 days with E2 (10 nM) and P4 (100 nM), ± FGF7 (50 ng/ml), ± SP600125 (10 µM). **(C-D)** T47D cells were cultured in 3D Matrigel for 14 days with E2 and P4, ± FGF7, ± SU6656 (10 µM) or LY294002 (2 µM). Representative images were taken (scale bar: 100 µm) **(C)** and colony size (relative to CTR/non-treated conditions) **(D)** was analysed using ImageJ software. Data are presented as means ± SD (n=3), *p<0.05, **p<0.005 by Student’s *t* test.

**Supplementary Fig. S8 Activity of GSK3β is involved in regulation of JunB expression. (A-B)** CAMA-1 cells were treated with E2 (oestrogen, 10 nM) and P4 (progesterone, 100 nM) ± SB216763 (10 µM) **(A)** or LiCl (20 mM) **(B)** for 6 and 12h. JunB and GSK3β (S9) was analysed by Western blotting. The bar plots are showing densitometry for JunB and GSK3β (S9). Densitometry data are presented as means ± SD (n=3). **(C) JunB is required for FGF7-triggered abrogation of the negative effect of P4 on breast cancer (BCa) cell growth.** Colony formation assay for T47D and T47D shJunB cells treated for 10 days with E2 (10 nM) ± P4 (100 nM), ± FGF7 (50 ng/ml).
